# A repeat region from the *Brassica juncea HMA4* gene *BjHMA4R* is specifically involved in Cd^2+^ binding in the cytosol under low heavy metal concentrations

**DOI:** 10.1186/s12870-019-1674-5

**Published:** 2019-02-28

**Authors:** Jianwu Wang, Shuang Liang, Weiwei Xiang, Huiping Dai, Yizhong Duan, Furen Kang, Tuanyao Chai

**Affiliations:** 1 Shaanxi Key Laboratory of Ecological Restoration in Shanbei Mining Area, Yulin University, Yulin, 719000 Shaanxi China; 2 College of Life Science, University of Chinese Academy of Sciences, Beijing, China; 3 College of Biological Science and Engineering, Shaanxi University of Technology, Hanzhong, Shaanxi 723001 People’s Republic of China

**Keywords:** Heavy metal ATPase (HMA), Heavy metal tolerance, Cadmium, Zinc, Substrate specificity, Hyperaccumulation

## Abstract

**Background:**

HMA4 transporters are involved in the transport and binding of divalent heavy metals (Cd, Zn, Pb [lead] and Co [cobalt]). In general, as efflux pumps, HMA4 transporters can increase the heavy metal tolerance of yeast and *Escherichia coli*. Additional research has shown that the C-terminus of HMA4 contains a heavy metal-binding domain and that heterologous expression of a portion of peptides from this C-terminal domain in yeast provides a high level of Cd tolerance and Cd hyperaccumulation.

**Results:**

We cloned *BjHMA4* from *Brassica juncea*, and quantitative real-time PCR analysis revealed that *BjHMA4* was upregulated by Zn and Cd in the roots, stems and leaves. Overexpression of *BjHMA4* dramatically affects Zn/Cd distribution in rice and wheat seedlings. Interestingly, BjHMA4 contains a repeat region named BjHMA4R within the C-terminal region; this repeat region is not far from the last transmembrane domain. We further characterized the detailed function of BjHMA4R via yeast and *E. coli* experiments. Notably, BjHMA4R greatly and specifically improved Cd tolerance, and BjHMA4R transformants both grew on solid media that contained 500 μM CdCl_2_ and presented improved Cd accumulation (approximately twice that of wild-type [WT] strains). Additionally, visualization via fluorescence microscopy indicated that BjHMA4R clearly localizes in the cytosol of yeast. Overall, these findings suggest that BjHMA4R specifically improves Cd tolerance and Cd accumulation in yeast by specifically binding Cd^2+^ in the cytosol under low heavy metal concentrations. Moreover, similar results in *E. coli* experiments corroborate this postulation.

**Conclusion:**

BjHMA4R can specifically bind Cd^2+^ in the cytosol, thereby substantially and specifically improving Cd tolerance and accumulation under low heavy metal concentrations.

**Electronic supplementary material:**

The online version of this article (10.1186/s12870-019-1674-5) contains supplementary material, which is available to authorized users.

## One-sentence summary

BjHMA4R can specifically bind Cd^2+^ in the cytosol, thereby substantially and specifically improving Cd tolerance and accumulation under low heavy metal concentrations.

## Background

*Brassica juncea*, commonly known as Indian mustard, is a species of mustard plant. According to U’s triangle, *B. juncea* (AABB genome, 2n = 4× = 36) is the allotetraploid derived from the diploid species *B. rapa* (AA, 2n = 2× = 20) and *B. nigra* (BB, 2n = 2× = 16) and arose by natural hybridization and chromosome doubling [1, 2]. This mustard plant can be used in phytoremediation to remove heavy metals and metalloids, such as cadmium (Cd), lead (Pb), zinc (Zn), cobalt (Co), nickel (Ni), copper (Cu), chromium (Cr), arsenic (As), cesium (Cs), and uranium (U), from the soil in hazardous waste sites. Thus, Indian mustard (*Brassica juncea*), a high-biomass-producing plant, has been broadly used as a model system to investigate the physiology and biochemistry of metal and radionuclide accumulation in plants [3–6]. However, the molecular mechanism of the hyperaccumulation phenotype remains unclear. HMA4 may play a significant role in the hyperaccumulation-related characteristics of hyperaccumulator species, at least within Brassicaceae plants.

HMA4 (heavy metal ATPase 4) is a member of the type 1_B_ heavy metal-transporting P-type ATPase subgroup, whose members transport the divalent cations Cd, Pb (lead), Zn, and Co (cobalt) [7, 8]. To date, this divalent class of transports exists only in prokaryotes and plants. There are four members in *Arabidopsis*, HMA1, HMA2, HMA3, and HMA4. AtHMA2 and AtHMA4 both have long C-terminal hydrophilic extensions that seem specific to plant P_1B_-ATPases [9, 10], and the C-terminal extension of AtHMA4, which includes 44 cysteine (Cys) and a terminal stretch of 11 consecutive histidine residues, is longer than that of AtHMA2. Moreover, the N-terminal regions of AtHMA2 and AtHMA4 are highly similar, and they exhibit similar expression patterns (predominantly in the vascular tissues of roots, stems and leaves) and subcellular localization (the plasma membrane) [11].

The sequence of TcHMA4, which is an ortholog of AtHMA4 from *Thlaspi caerulescens*, is 76% similar to that of AtHMA4, has more divergent C-teminal sequence and shares only 43% identity with the counterpart in AtHMA4. Interestingly, two truncated peptides from the TcHMA4 C-terminal domain were expressed in yeast, which resulted in an extremely high level of Cd tolerance and hyperaccumulation [12–14]. There are 3 *HMA4* gene copies in the *Arabidopsis halleri* genome, and the activity of all three *AhHMA4* promoters is much higher than the *AtHMA4* promoter activity, leading to the high *HMA4* transcript levels in *A. halleri*. Moreover, QTL analysis, physiology experiments and reverse genetic studies have demonstrated that *AhHMA4* has played a major role in the natural selection of Zn hyperaccumulation and associated Cd and Zn hypertolerance in *A. halleri* [15].

Taken together, the striking differences in gene coding sequence, especially at the 3′-end, *cis*-regulatory regions and gene copy numbers observed between the hyperaccumulator *T. caerulescens* and *A. halleri* and *A. thaliana*, the latter of which is a close nontolerant nonaccumulator relative, suggest that *HMA4* was subjected to positive selection during adaptive evolution. These differences led to changes in *HMA4* expression levels and physiological function predominantly in substrate specificity and C-terminal heavy metal binding capacity. Generally, HMA4s have a conserved N-terminus and more divergent C-terminus. The functional domains of the N-terminus have been well characterized by sequence alignments and experiments, but the positions and functions of the C-terminal domains have not been well studied.

In the present study, we cloned *BjHMA4* from *B. juncea*. Compared with other HMA4s, BjHMA4 had a more divergent and long C-terminal hydrophilic extension and repeat region (named BjHMA4R, which had 4 CC dipeptides [Cys pairs]). We then characterized BjHMA4R in detail to investigate its function. Our results show that BjHMA4R facilitates a high degree of heavy metal tolerance in yeast via its high binding activity and possibly interacts with other heavy metal transporters as a chaperone, resulting in increased heavy metal flux.

## Results

### Characterization of *BjHMA4*

#### Isolation of *B. juncea* HMA4 cDNA

**Fig. 1:**
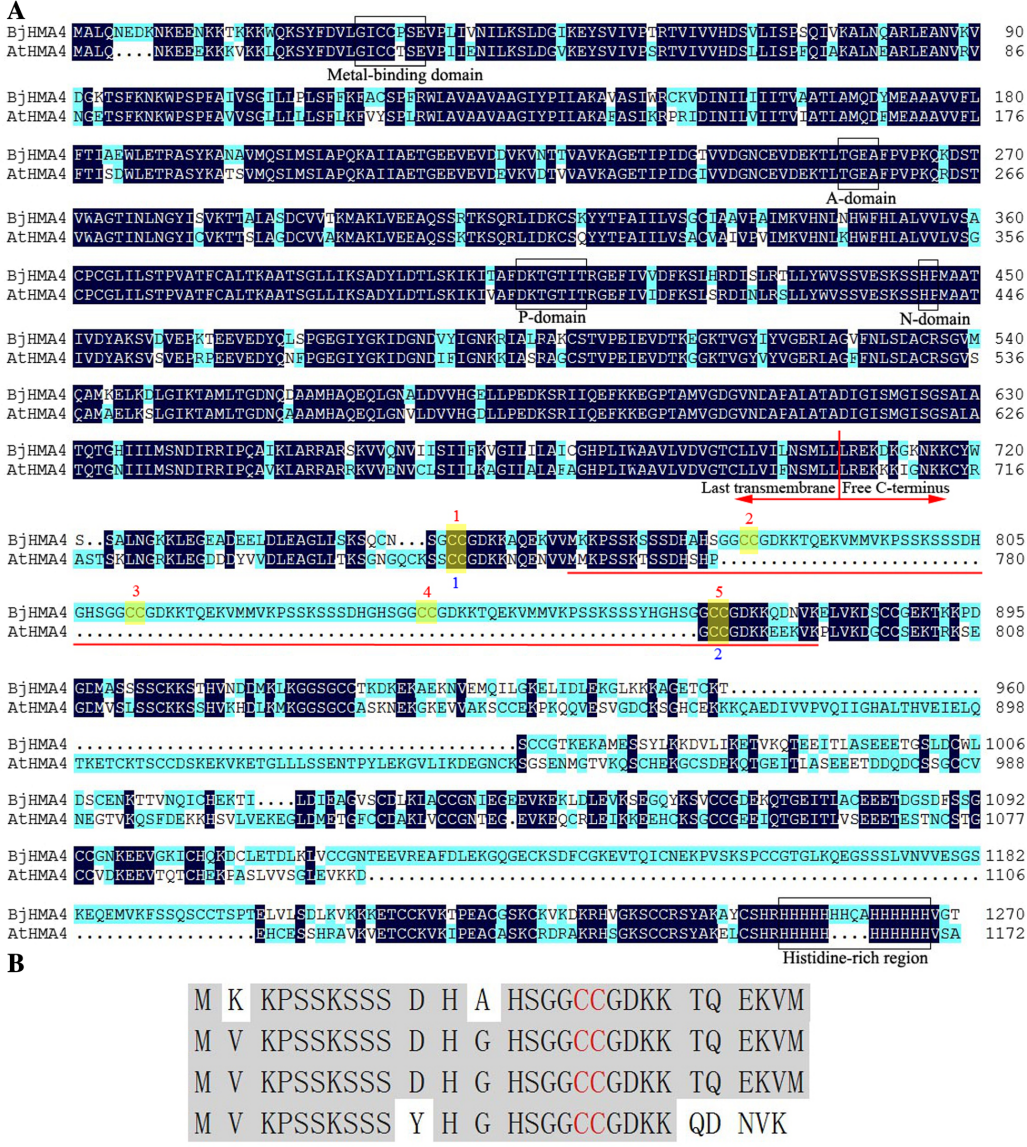
**a** Sequence alignment of BjHMA4 from *B. juncea* and the *Arabidopsis* homolog AtHMA4. The deduced amino acid sequences of BjHMA4 and AtHMA4 (accession No. 064474) shown were aligned using DNAMAN. The dark blue shading indicates identical residues. Several domains common to P_1B_-type ATPases are indicated by boxes. Additionally, the numerous Cys residue pairs within the C-terminal region are indicated by numbers. The repeat region in BjHMA4 (BjHMA4R) is underlined in red. **b** The repeat region in BjHMA4 (BjHMA4R). Cys residue pairs are shown in red

Based on homology cloning strategies and the RACE technique, the *BjHMA4* gene cloned from *B. juncea* was submitted to GenBank (accession No. JQ673430) and was found to encode 1271 amino acids and have six transmembrane domains (predicted using the TMHMM 2.0 server, http://www.cbs.dtu.dk/services/TMHMM/). The deduced amino acid sequence of BjHMA4 is 61 and 64% identical to that of AtHMA4 (from *A. thaliana*) and TcHMA4 (from *T. caerulescens*), respectively. The low sequence similarity was due to the high divergence of the BjHMA4-C region, i.e., the sequence after the last transmembrane domain. Alignment with AtHMA4 indicated that BjHMA4 contains 5 highly conserved domains of HMA4: the metal-binding domain (binds heavy metals), A-domain (which includes the smaller cytoplasmic loop region; this domain functions as the ‘actuator’ of the gating mechanism that regulates Ca^2+^ binding and release), P-domain (which is homologous to members of the catalytic HAD family of enzymes; this domain contains the phosphorylation site), N-domain (contains the ATP-binding site) and histidine-rich region (which may also participate in heavy metal binding) (Fig. 1a) [16]. Interestingly, the BjHMA4 C-terminal region contains an apparent 4-repeat region (named BjHMA4R) that is not far from the last transmembrane domain (Fig. 1a), which increases the number of CC dipeptides (Cys pairs) in BjHMA4 (AtHMA4 contains 13, TcHMA4 contains 16, and BjHMA4 contains 17) (Fig. 1b).

#### Expression profiles of *BjHMA4*

**Fig. 2:**
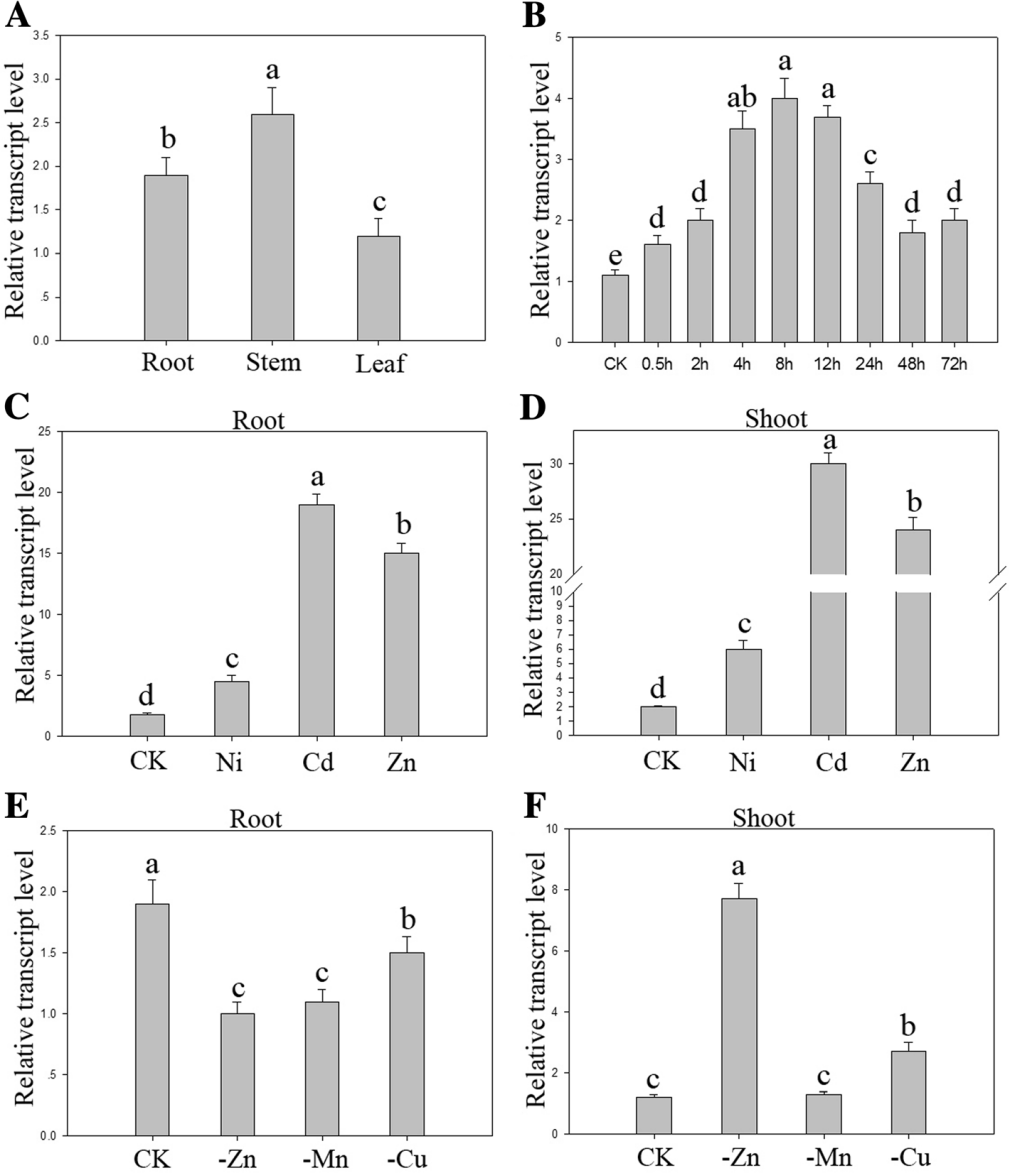
Expression profiles of *BjHMA4* in *B. juncea*. **a** Expression analyses of *BjHMA4* genes under normal conditions. The total RNA was extracted from the roots, stems, and leaves of three-week-old *B. juncea* plants. **b** The expression levels of *BjHMA4* in the roots under different ZnCl_2_ treatment periods. **c**, **d** Expression analyses of *BjHMA4* genes in the roots and shoots in response to heavy metal treatments. Three-week-old WT *B. juncea* plants were exposed to 400 μM NiCl_2_, 200 μM CdCl_2_, or 2000 μM ZnCl_2_ for 6 h. **e**, **f** Expression analyses of *BjHMA4* genes in the roots and shoots under Mn-, Cu- or Zn-deficient conditions. Two-week-old *B. juncea* plants were transferred to Mn-, Cu- or Zn-depleted media for one week. The data represent the means of three biological replicates. Different letters above the columns indicate significant differences (*P* < 0.05) between seedling groups under the same stress treatment

The expression of *BjHMA4* was studied *in planta* via quantitative real-time PCR analysis under stringent conditions. The primers used were designed from the 3` region of the cDNA, which was predicted to be the most divergent among the different *HMAs*. There was no significant difference in *BjHMA4* expression among the roots, stems and shoots at the seedling stage; however, the expression in the roots increased upon treatment with 600 μM ZnCl_2_ and subsequently decreased to normal levels depending on the treatment duration. The level of BjHMA4 transcripts was upregulated and peaked after 8 h of treatment; this level was approximately four times greater than normal levels. The BjHMA4 expression in the roots and shoots increased approximately 10–20-fold under Zn and Cd stresses and slightly increased under Ni stress. Under Zn, Mn, Cu deficiency stresses, BjHMA4 expression increased approximately 4-fold under Zn deficiency stress only in the shoots (Fig. 2).

### Overexpression of *BjHMA4* only slightly increases Zn/cd tolerance in yeast

**Fig. 3:**
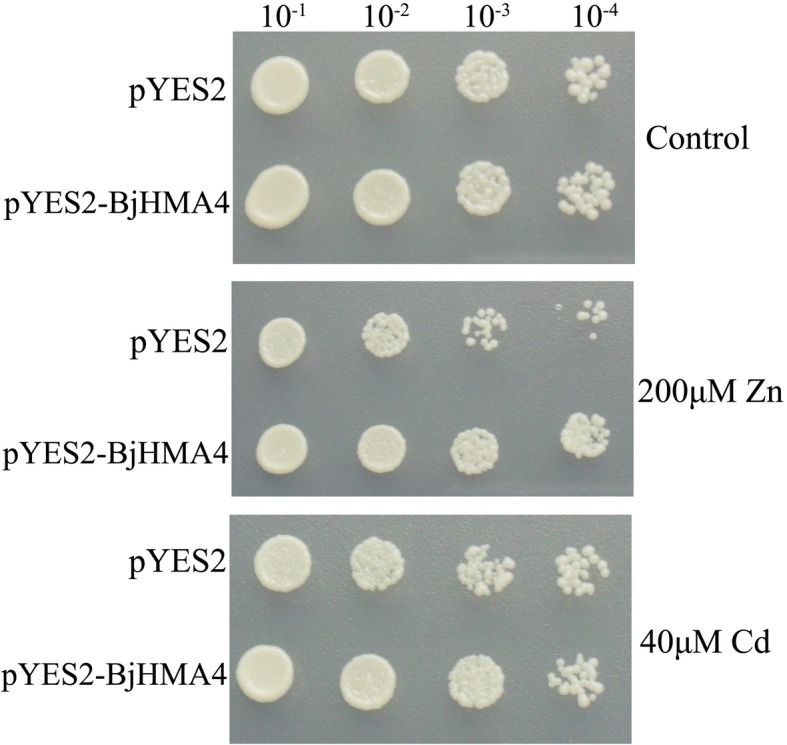
Zn and Cd tolerance of *BjHMA4*-transformed yeast (YK44) cells. YK44 (ura3–52 his3–200, △ZRC, △Cot1, mating type a) lacks the ability to accumulate Zn/Cd in vacuoles, and pYES2-*BjHMA4*-transformed YK44 grows slightly better than the control in the presence of 200 μM ZnCl_2_ and 40 μM CdCl_2_

The pYES2 and pYES2-BjHMA4 vectors were transformed into yeast YK44 (ura3–52 his3–200, △ZRC, △Cot1, mating type a), which is sensitive to Zn and Cd, to investigate whether BjHMA4 could transport and bind these metals. We observed that compared with the empty vector, the pYES2-BjHMA4-transformed YK44 grew fairly well on YPD plates containing 200 μM ZnCl_2_ and 40 μM CdCl_2_ (Fig. 3).

### Overexpression of *BjHMA4* dramatically affects Zn/cd distribution in rice and wheat seedlings

**Fig. 4:**
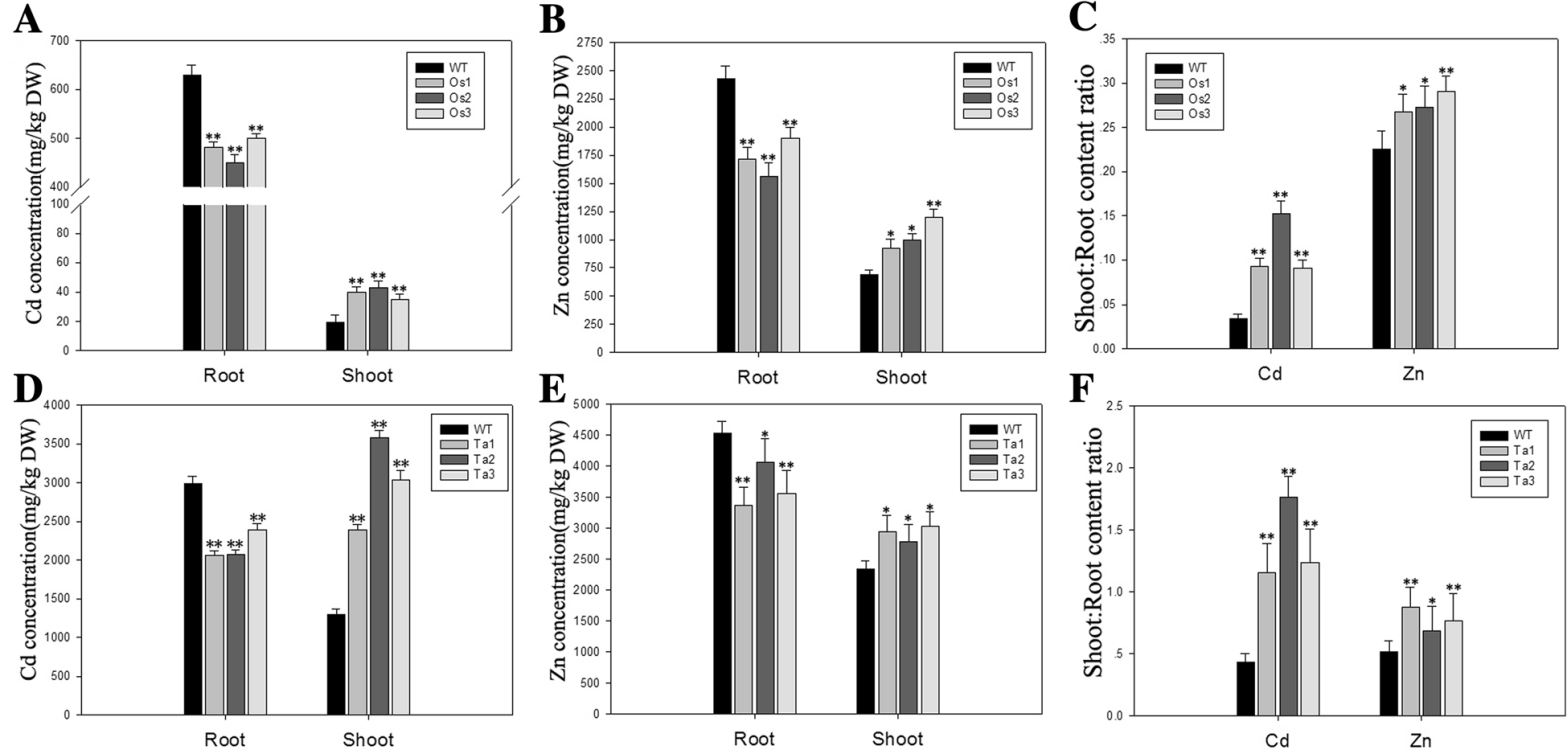
The Zn/Cd content in the *BjHMA4* transgenic and corresponding wild-type (WT) plants. Shown are cadmium (**a**, **d**) and zinc concentrations (**b**, **e**) in the roots and the shoots and the shoot:root ratios (**c**, **f**) of cadmium and zinc concentrations in 3-week-old rice and wheat transformed with *BjHMA4* and WT rice and wheat grown hydroponically for 2 weeks and subsequently exposed to 200 μM CdCl_2_ or 2 mM ZnCl_2_ for a week. The data are expressed as the mean ± SE of three replicates; * and ** indicate significance at 5 and 1% levels (evaluated by Student’s *t* test), respectively

The results of semi-reverse transcription-PCR and real-time PCR analysis verified BjHMA4 expression in the transgenic rice and wheat (Additional file 1: Data S1). The overexpression of BjHMA4 in rice and wheat, which was driven by the ubiquitin and Actin1 promoter respectively, affected the Zn/Cd distribution of the transgenic plants. The Zn and Cd contents decreased significantly in the roots but increased in the shoots of the transgenic rice and wheat. Thus, the root-shoot Zn/Cd translocation significantly increased in BjHMA4 transgenic rice and wheat (Fig. 4). These results suggested that BjHMA4 may function as an efflux pump in the vascular tissues.

### Characterization of *BjHMA4R*

#### Homologous sequences of BjHMA4R only exist in Brassicaceae

**Fig. 5:**
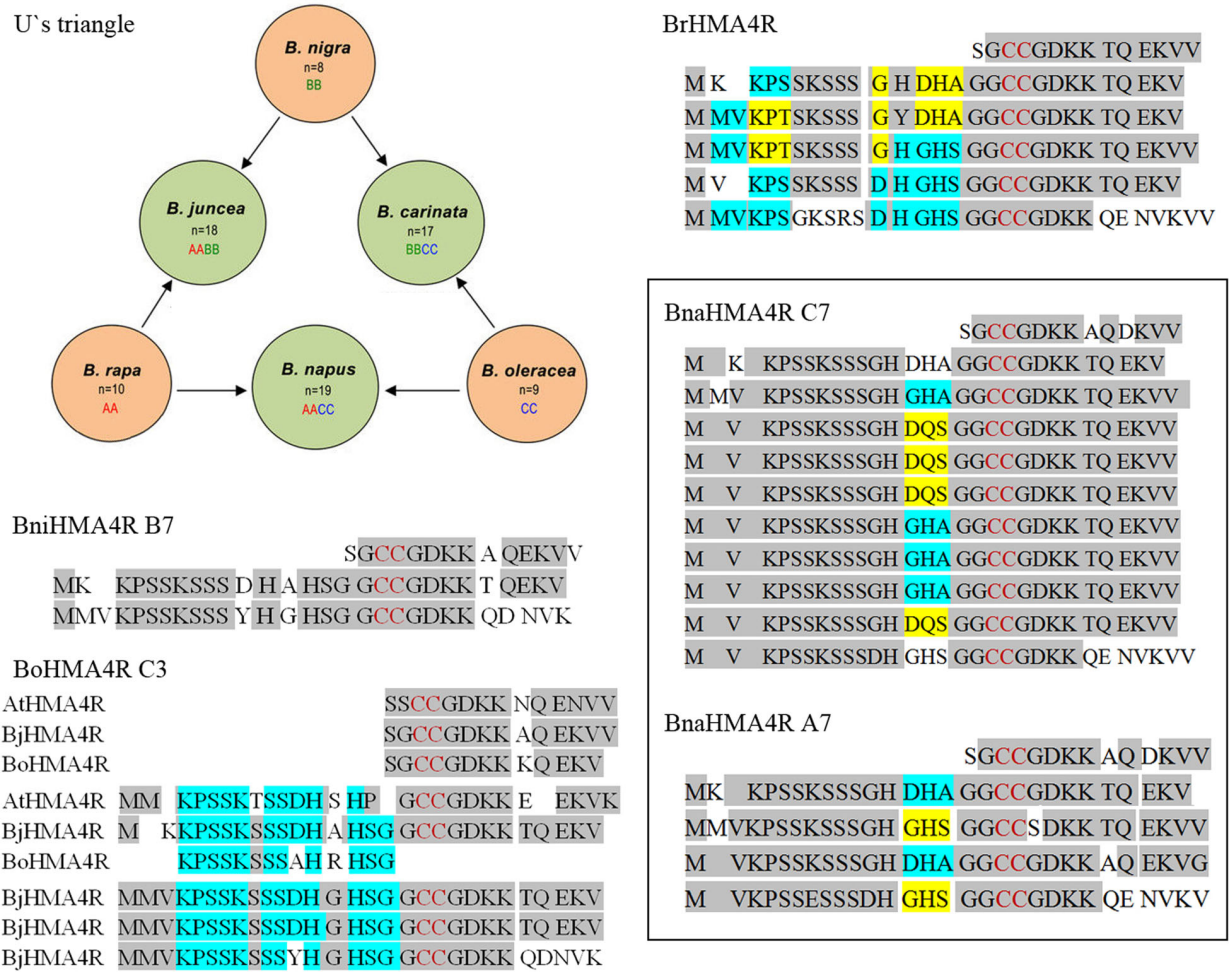
Homologous sequences of BjHMA4R in U’s triangle plants. BniHMA4R indicates HMA4R from *B. nigra*, BoHMA4R indicates HMA4R from *B. oleracea*, BrHMA4R indicates HMA4R from *B. rapa*, and BnaHMA4R indicates HMA4R from *B. napus*. A7, B7, C3, and C7 indicate chromosome numbers

To determine whether other plants possess homologous sequences to BjHMA4R, we used the *BjHMA4* cDNA sequence as a querying probe to search against other plant genomes in GenBank by the online BLAST tool (https://blast.ncbi.nlm.nih.gov/Blast.cgi). The results showed that highly homologous sequences existed only in Brassicaceae. *Carica papaya* (genome taxid: 3649), which belongs to Brassicales, has only an N-terminal homologous sequence to BjHMA4, not C-terminal. Similar results were obtained from *Medicago truncatula* (taxid: 3880), *Solanum lycopersicum* (taxid: 4081), and *Nicotiana tabacum* (taxid: 4907). Interestingly, plants that belong to *Brassica*, especially the 6 species that belong to U’s triangle, had highly homologous sequences. Among the 3 diploids, *B. nigra* (BB, genome taxid: 3710) had 2 repeats located on chromosome B7 (sequence ID: CM004497.1), *B. rapa* (AA, taxid: 3711) had 5 repeats located on chromosome A7 (sequence ID: NC_024801.1), and *B. oleracea* (CC, taxid: 3712) had a partial repeat located on chromosome C3 (sequence ID: NC_027750.1) (Fig. 5). Among the 3 allotetraploids, only *B. napus* had homologous sequences in (AACC, genome taxid: 3708): chromosome A7 (sequence ID: NC_027763.2) had 4 repeats, and chromosome C7 (sequence ID:NC_027773.2) had 10 repeats. We speculate that *B. juncea* (AABB, taxid: 3707) and *B. carinata* (BBCC, taxid: 52824) showed no homologous sequences because their genome sequences have not been fully assembled yet (Fig. 5). *Arabidopsis thaliana* and *Arabidopsis halleri* both had 1 repeat. TcHMA4 had 1 repeat, which had more different amino acid residues than the others. There are 6 strongly conserved amino acid residues (CCGDKK) in the Brassicaceae plants as analyzed here. The CC in that sequence is the first cystine pair in the C-terminus, and this six-residue sequence is part of the repeat and is at the front of the repeats in the Brassicaceae plants that we analyzed (Fig. 5 and Additional file 2: Data S2) .

#### Subcellular localization of BjHMA4R

**Fig. 6:**
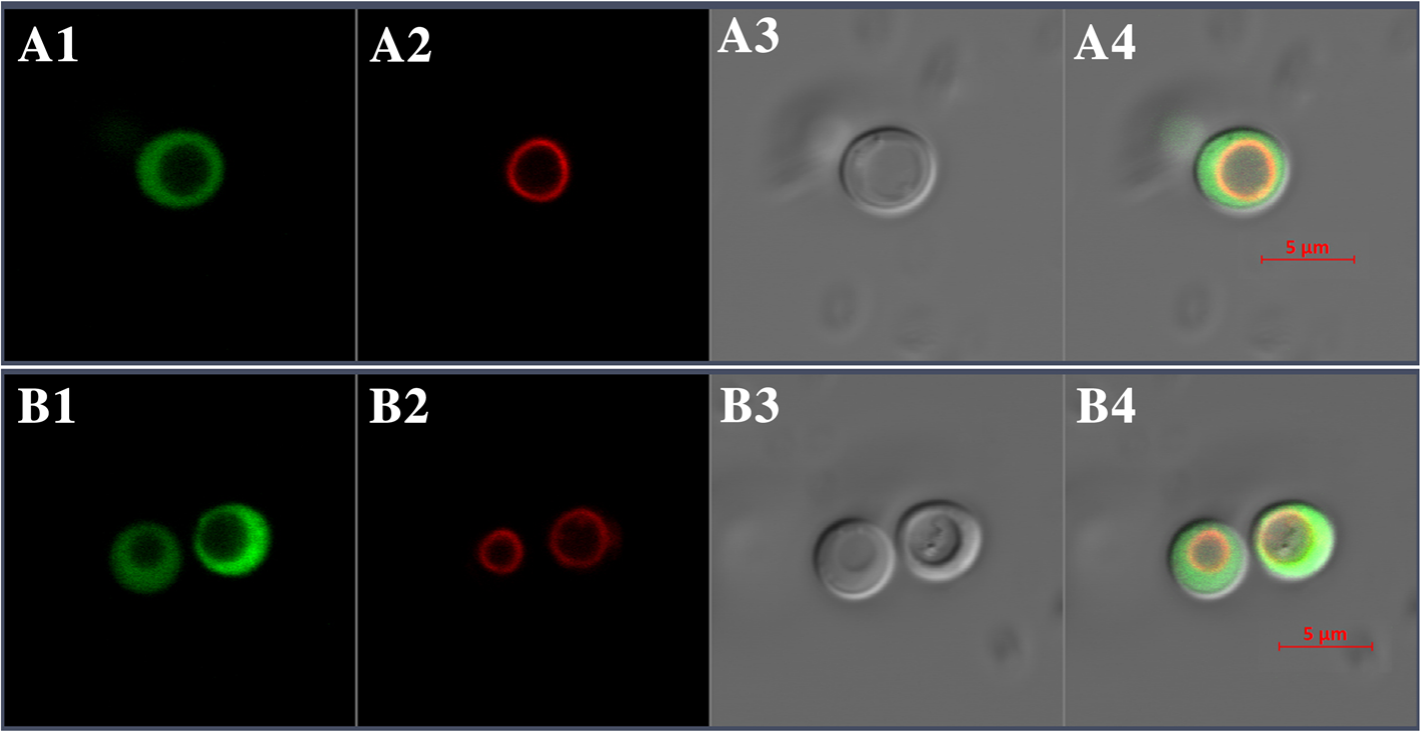
Subcellular localization of pUG36 (A1-A4) and pUG36-BjHMA4R (B1-B4) fusion proteins in yeast strain BY4741. Yeast cells expressing pUG36 and pUG36-BjHMA4R were cultured in SD-URA media supplemented with Glu; however, when the cells reached the stationary phase, they were then transferred to SD-URA-Met media supplemented with Glu for 12 h. A1 and B1, eGFP fluorescence signals; A2 and B2, vacuoles; A3 and B3, bright-field images; A4 and B4, overlay of GFP fluorescence signals, vacuoles and bright-field images. Bars = 5 μm

BjHMA4R clearly localizes to the yeast cytosol. When cells in the stationary phase were analyzed, green fluorescence from the expression of pUG36-BjHMA4R was distributed throughout the cytosol. To verify the localization of the fusion protein in the cytosol, the vacuolar membrane was stained for a short period of time with the lipophilic dye FM4–64, which selectively stains the yeast vacuolar membrane. The merged GFP and FM4–64 images demonstrated that GFP-tagged BjHMA4R was localized in the yeast cytosol (Fig. 6). Different yeast strains, including BY4741 and *end3* (data not shown), were used to determine the subcellular localization of BjHMA4R, and the same result was obtained with both strains. This result is consistent with that via the bioinformatics-based hydropathy analysis method.

### Expression of *BjHMA4R* in yeast and *E. coli* provides heavy metal tolerance

**Fig. 7:**
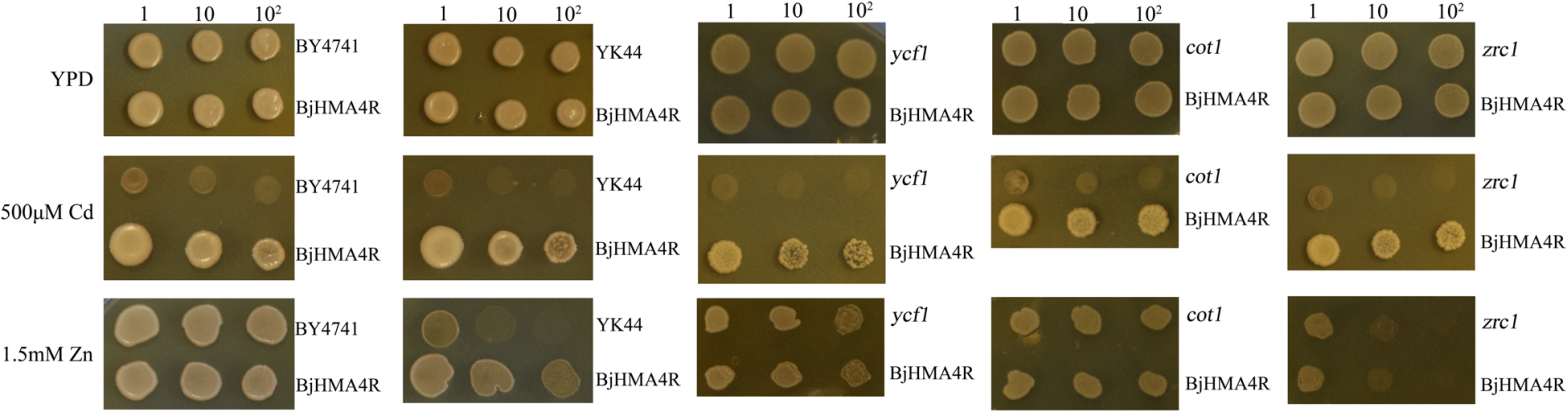
Growth of yeast cells expressing *BjHMA4R* under Cd stress. BY4741, YK44, *ycf1*, *cot1* and *zrc1* transformants expressed pYES2 (negative control) and *BjHMA4R*, respectively. The cultures were adjusted to an OD_600_ of 1 and were serially diluted 10-fold in water. Five-microliter aliquots of each dilution were spotted either on nonselective YPD plates or on YPD plates supplemented with 500 μM CdCl_2_ and 1.5 mM ZnCl_2_. After 3 days of incubation at 30 °C, the plates were imaged. The dilutions are indicated in the above figure, and three individual clones of each yeast transformant were analyzed

**Fig. 8:**
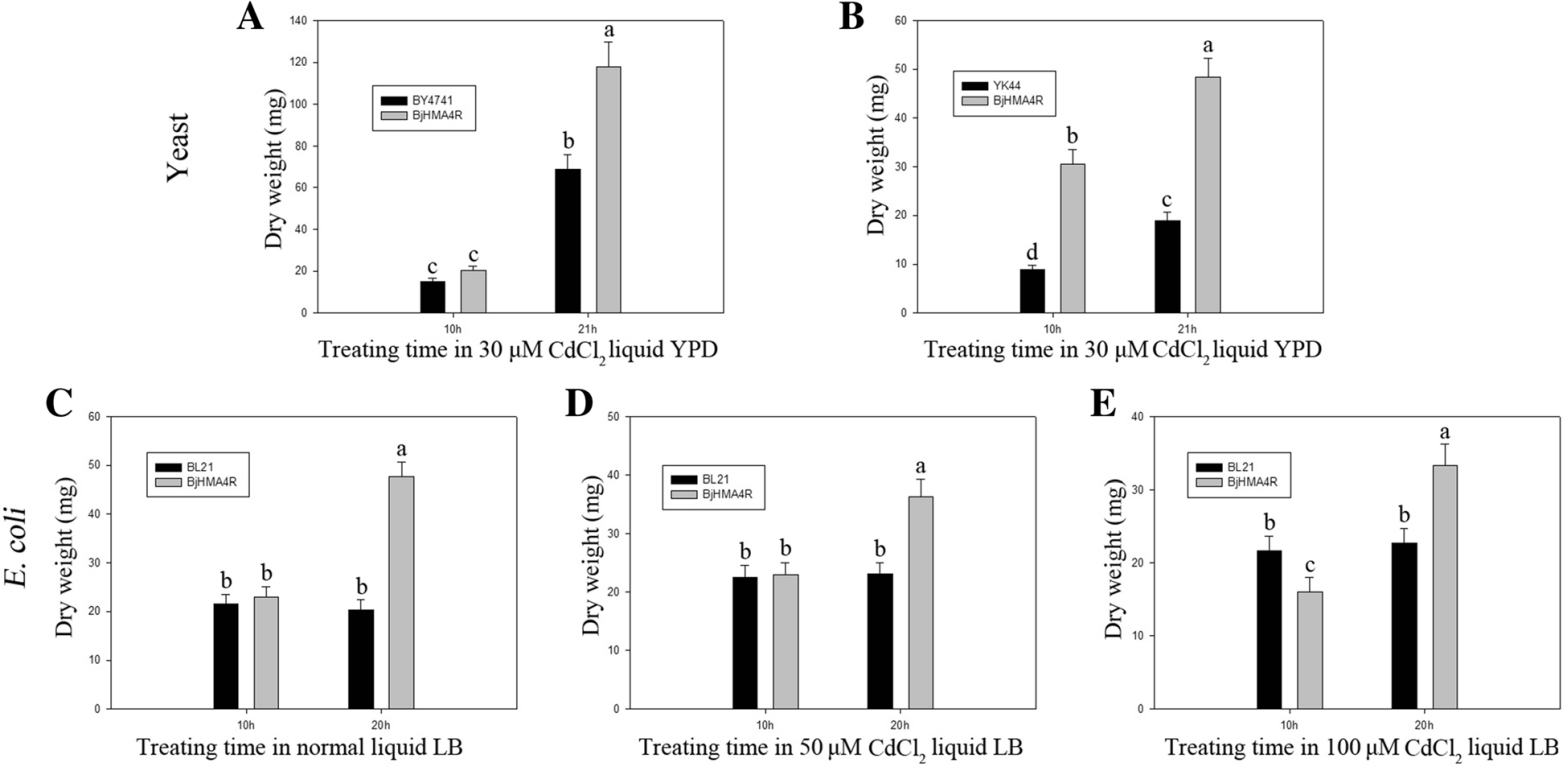
Dry weight of yeast and *E. coli* expressing *BjHMA4R*. **a**, **b** Yeast BY4741 and YK44 cells transformed with a pYES2 plasmid and a pYES2 plasmid that harbored *BjHMA4R* were grown in liquid YPD medium supplemented with 30 μM CdCl_2_. The cells were incubated at 30 °C for 10 h and 21 h. Fifteen milliliters of yeast fluid was collected by centrifugation and then dried at 95 °C for 48 h for determination of the dry weight. **c**, **d**, **e** *E. coli* BL21 cells transformed with a pEASY-Blunt E1 expression plasmid or with a pEASY-Blunt E1 expression plasmid that harbored *BjHMA4R* were grown in liquid LB medium supplemented with normal LB, 50 μM CdCl_2_ and 100 μM CdCl_2_. The cells were incubated at 37 °C for 10 h and 20 h. Fifteen milliliters of *E. coli* fluid was collected by centrifugation and then dried at 95 °C for 48 h to determine the dry weight. The results are the means ± SEs of four independent experiments performed with four different colonies. Different letters above the columns indicate significant differences (*P* < 0.05) between different cell groups under the same stress treatment

To investigate the substrate specificity of BjHMA4R and its functional properties, the protein was expressed in BY4741 (WT), *ycf1*, *zrc1*, *cot1* and *zrc1&cot1* (YK44) yeast strains. All the strains were transformed with *BjHMA4R*, resulting in a significant level of Cd tolerance but not Zn, Ni, Co or Pb tolerance (Additional file 3: Data S3). As shown in Fig. 7, the growth of the BY4741 and YK44 cells expressing the empty pYES2 vector was strongly inhibited by the inclusion of 500 μM Cd in the solid YPD media, while compared with control cells, yeast cells expressing *BjHMA4R* grew significantly more on substantially high levels of Cd (500 μM) in solid YPD media and grew better in liquid YPD media that contained 30 μM Cd (Fig. 8). The dry weight of the BY4741 and YK44 transformants expressing BjHMA4R were both significantly higher than that of the control cells after 21 h of culture, and the dry weight increased approximately 1-fold (Fig. 8). These findings indicated that the growth superiority of yeast transformants with *BjHMA4R* was apparent under Cd stress, particularly for the Cd-sensitive YK44 double mutant. In yeast, ZRC1 and COT1 are members of the CDF cation transporter family and are localized in the vacuolar membrane. ZRC1 is mainly responsible for transporting Zn^2+^ and Cd^2+^, and COT1 is mainly responsible for transporting Co^2+^ from the cytosol into the vacuole [17]. Thus, the YK44 double mutant (*cot1&zrc1*) is sensitive to Zn, Cd and Co stress. The results of this experiment showed that the substrate specificity of ZRC1 and COT1 was not very high, and the *zrc1* and *cot1* strains were not sensitive to Zn under 1.5 mM Zn stress; however, the YK44 strain was sensitive to Zn. Interestingly, BjHMA4R-transformed YK44 could rescue the Zn sensitivity phenotype of YK44 under 1.5 mM Zn stress (Fig. 7).

**Fig. 9:**
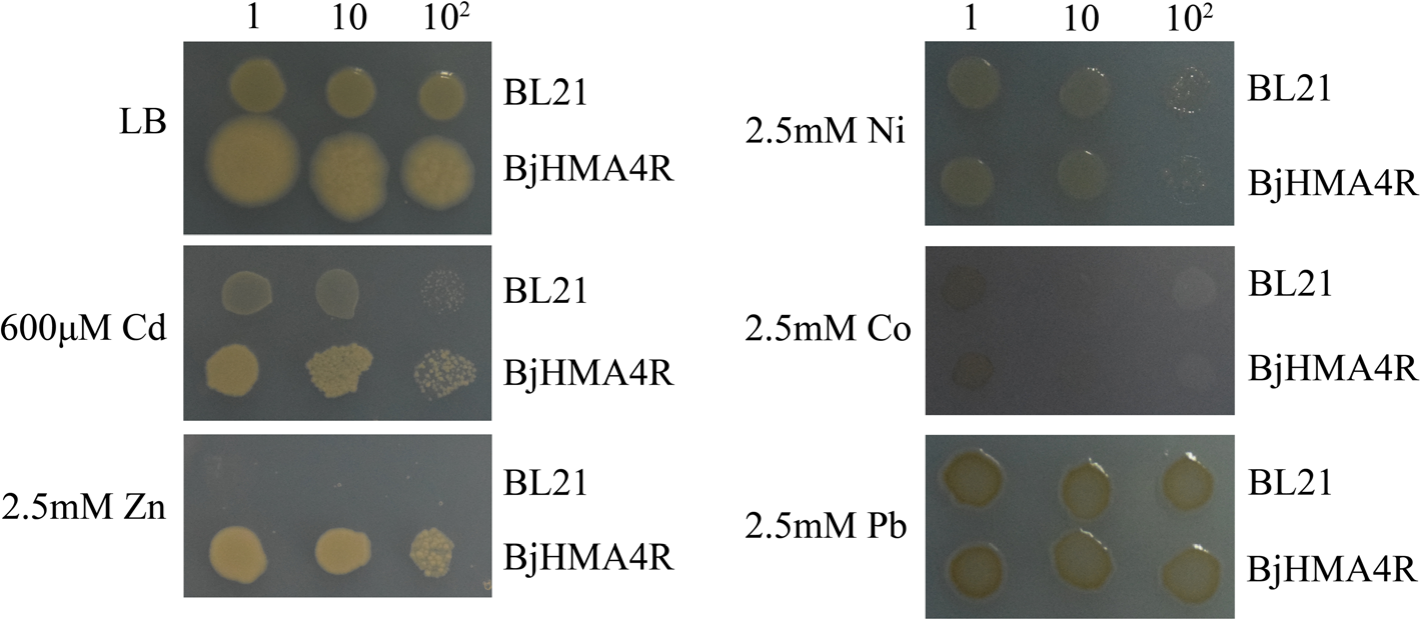
Growth of *E. coli* expressing *BjHMA4R*. BL21 transformants contained a pEASY-Blunt E1 expression vector (negative control) and *BjHMA4R*, respectively. The cultures were adjusted to an OD_600_ of 1 and were serially diluted 10-fold in water. Five-microliter aliquots of each dilution were spotted either on nonselective LB plates or on LB plates supplemented with 600 μM CdCl_2_ and 2.5 mM ZnCl_2,_ Ni(NO3)_2_, Co(NO3)_2_ and Pb(NO3)_2_. After 1 day of incubation at 37 °C, the plates were imaged. The dilutions are indicated in the above figure, and three individual clones of each *E. coli* transformant were analyzed

Similar results were obtained from our *E. coli* experiments. Compared with control cells, strain BL21 cells transformed with BjHMA4R exhibited significant levels of Cd (600 μM) and Zn (2.5 mM) tolerance and greater growth in solid LB media but not Ni, Co or Pb tolerance (Fig. 9). The dry weights of the BL21 transformants expressing BjHMA4R were significantly higher than those of the control cells after 20 h of culture, especially when cultured in normal and 50 μM CdCl_2_-supplemented liquid LB media (Fig. 8).

### Heavy metal accumulation in control cells and BjHMA4R-transformed yeast and *E. coli* cells

**Fig. 10:**
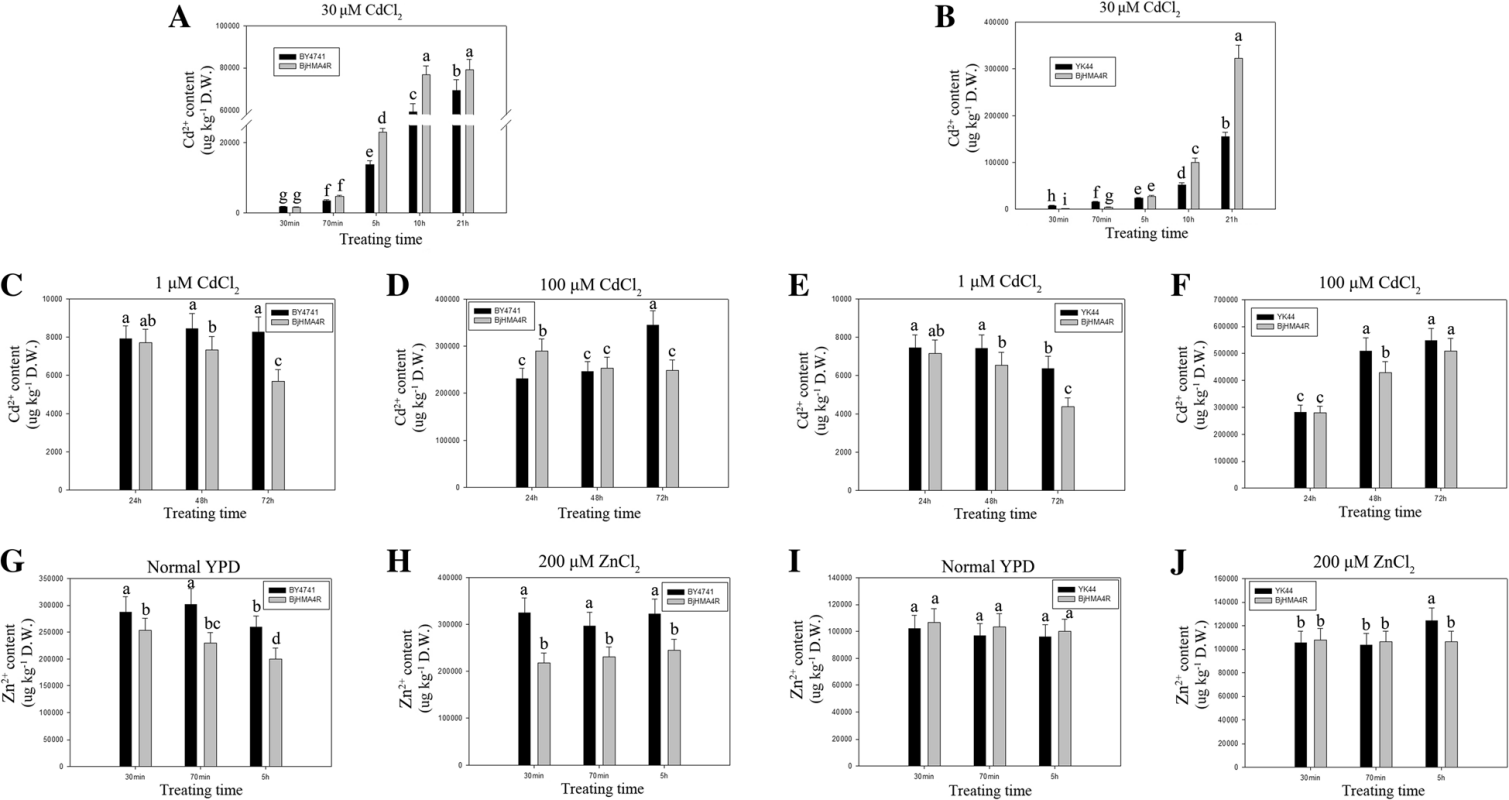
Cd and Zn contents of yeast expressing *BjHMA4R*. Yeast BY4741 and YK44 cells transformed with a pYES2 plasmid or a pYES2 plasmid that harbored *BjHMA4R* were grown in normal liquid YPD medium overnight. Then, they were supplemented with 30 μM CdCl_2_ (**a**, **b**), 1 μM CdCl_2_ (**c**, **e**), 100 μM CdCl_2_ (**d**, **f**), normal medium (**g**, **i**), or 200 μM ZnCl_2_ (**h**, **j**). The cells were incubated at 30 °C for 30 min, 70 min, 5 h, 10 h or 21 h. The metal contents of the samples were analyzed with inductively coupled plasma-mass spectrometry (ICP-MS). The results are the means ± SEs of four independent experiments performed with four different colonies. Different letters above the columns indicate significant differences (*P* < 0.05) between cell groups under the same stress treatment

Metal accumulation experiments were conducted with WT yeast (BY4741) and double mutant yeast (YK44) cells expressing empty pYES2 vectors as well as yeast cells expressing *BjHMA4R*. These experiments involved both yeast strains and genotypes incubated in liquid YPD medium supplemented with either 30 μM CdCl_2_ or 200 μM ZnCl_2_ and harvesting yeast cells after 30 min, 70 min, 5 h, 10 h, and 21 h of metal accumulation. In general, the metal accumulation profiles of the BY4741 and YK44 strains distinctly differed from those of the control and the *BjHMA4R*-transformed yeast strain at different treatment time points (Fig. 10a, b). As shown in Fig. 10, *BjHMA4R*-transformed BY4741 always accumulated more Cd than did BY4741 at every time point, and the maximum difference emerged after 5 h of treatment. *BjHMA4R*-transformed BY4741 accumulated approximately 70% more Cd than did the control. With respect to YK44, *BjHMA4R*-transformed YK44 accumulated more than 4-fold less Cd than did the control YK44 at the 30 min time point and 2.7-fold less Cd at 70 min. However, *BjHMA4R*-transformed YK44 accumulated more Cd than did the control YK44 after 5 h of treatment, and the difference was more clear. *BjHMA4R*-transformed YK44 accumulated approximately twice as much Cd as did the control YK44 after 21 h of treatment (Fig. 10b). There was no apparent difference in Cd content between the *BjHMA4R*-transformed and control strains under low and high concentrations of Cd (1 and 100 μM CdCl_2_) and long periods of stress (24 h, 48 h, and 72 h) (Fig. 10c, d, e, f). There was no significant difference in Zn accumulation between the *BjHMA4R*-transformed cells and control cells under 200 μM ZnCl_2_ stress, but the Zn accumulation apparently differed between BY4741 and YK44; BY4741 accumulated approximately 1.5-fold more Zn than did YK44(Fig. 10g, h, i, j). Similar results were obtained by 200 μM Ni(NO3)_2_, Co(NO3)_2_, and Pb(NO3)_2_ stress treatments. BY4741 accumulated more Co and less Ni than did YK44, but the Pb content was about the same (Additional file 4: Data S4**a**, **b**, **d**, **e**, **g**, **h**).

**Fig. 11:**
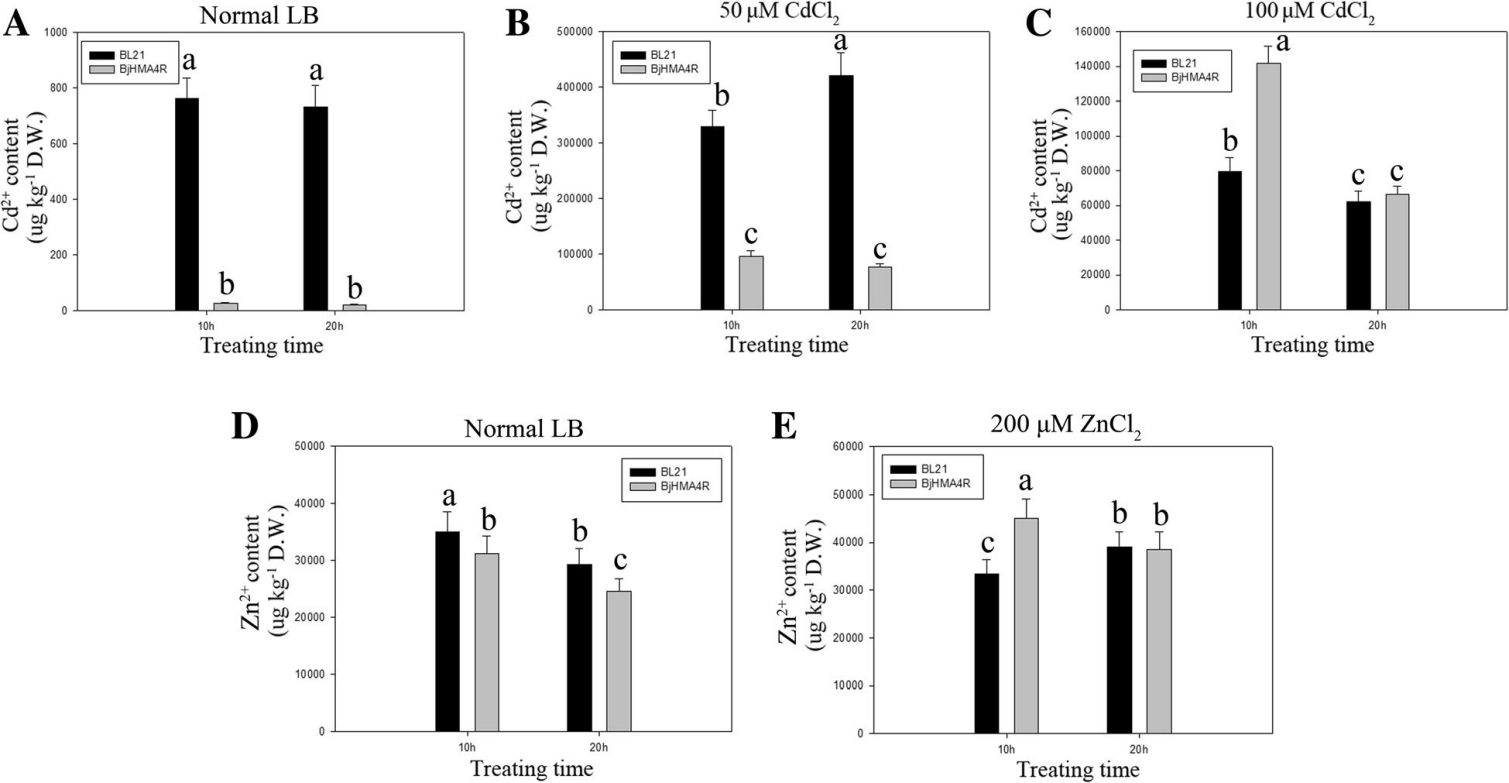
Cd and Zn contents of *E. coli* expressing *BjHMA4R*. *E. coli* BL21 cells transformed with a pEASY-Blunt E1 expression plasmid or a pEASY-Blunt E1 expression plasmid that harbored *BjHMA4R* were grown in normal liquid LB medium overnight, then supplemented with 50 μM CdCl_2_ (**b**), 100 μM CdCl_2_ (**c**), 200 μM ZnCl_2_ (**e**) and normal LB (**a**, **d**). The cells were incubated at 37 °C for 10 h and 20 h. The metal contents of the samples were analyzed with inductively coupled plasma-mass spectrometry (ICP-MS). The results are the means ± SEs of four independent experiments performed with four different colonies. Different letters above the columns indicate significant differences (*P* < 0.05) between cell groups under the same stress treatment.

With respect to *E. coli*, *BjHMA4R*-transformed BL21 accumulated less Cd than did the control BL21 under normal culture conditions and under low-concentration Cd (50 μM CdCl_2_) stress(Fig. 11a, b). There was no apparent difference in Cd content between the *BjHMA4R*-transformed and control strains under high concentrations of Cd (100 μM CdCl_2_) and long periods of stress (21 h) (Fig. 11c). Interestingly, both BL21 genotypes (*BjHMA4R*-transformed and control ones) presented approximately 1-fold more Cd accumulation under 50 μM CdCl_2_ stress than under 100 μM CdCl_2_ stress (Fig. 11b, c). Zn accumulation of *BjHMA4R*-transformed BL21was slightly but significantly less than did the control BL21 under normal culture conditions(Fig. 11d), however, *BjHMA4R*-transformed BL21 had significantly more Zn than did the control under 200 μM ZnCl_2_ and relatively short periods of stress (10 h) (Fig. 11e). There were no significant differences in Ni, Co, or Pb accumulation between *BjHMA4R*-transformed and control BL21 cells under 200 μM Ni(NO3)_2_, Co(NO3)_2_, or Pb(NO3)_2_ stress (Additional file 4: Data S4**c**, **f**, **i**).

### Cd^2+^ flux from transformed yeast and *E. coli* cells in response to CdCl_2_ stress treatments

Using the NMT (Non-invasive Micro-test Technology) technique, we measured Cd^2+^ flux across cell membranes. In response to 30 μM CdCl_2_ stress, BjHMA4R changed the signatures of Cd^2+^ flux of transformed cells in comparison with control cells.

**Fig. 12:**
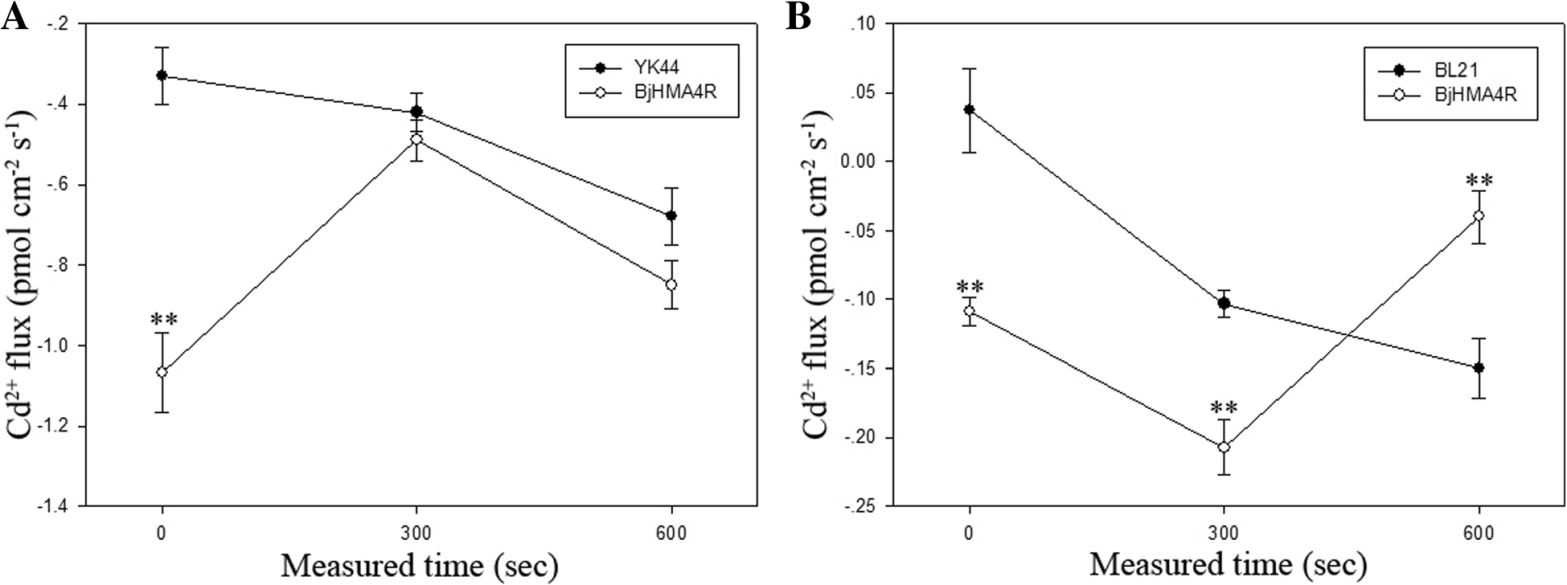
Detection of BjHMA4R activity by Cd^2+^ flux measurements. Transgenic BjHMA4R and control cells were exposed to 30 μM Cd^2+^ and measurements were taken for 600 s, using a vibrating probe, after the flux became ready. **a** yeast cells**, b** *E. coli* cells**.** The data are expressed as the mean ± SE of three replicates; * and ** indicate significant levels at 5 and 1% (evaluated by Student’s *t* test), respectively

Overexpression of *BjHMA4R* can increase Cd^2+^ influx of transgenic yeast and *E. coli* cells when just under Cd stress compared with control cells (Fig. 12). With the extension of treatment time, the Cd^2+^ flux of yeast cells tended to be consistent (Fig. 12a), and the transgenic cells of *E. coli* had significantly Cd^2+^ efflux compared with control cells (Fig. 12b). The above results are basically consistent with those of metal content analysis.

## Discussion

### HMA4 C-terminus may have important physiological functions

In their N-terminal regions, AtHMA4 and TcHMA4 present the degenerate MBD domain sequence G_25_ICCTSE_31_ [12]. This domain is very conservative, and all substitutions affecting this degenerate core sequence had deleterious effects on the AtHMA4 complementation efficiency [11]. However, the N-terminal MBD domain of BjHMA4 bears the sequence G_29_ICCPSE_35_ (Fig. 1); substitutions of T with P should not affect the activity of BjHMA4 because T and P have similar structures and chemical properties, and the R-groups of both are uncharged. The A-domain, P-domain and N-domain sequences are identical to those of AtHMA4 (Fig. 1). However, BjHMA4 bears a longer histidine-rich region than does AtHMA4 and TcHMA4; AtHMA4 and TcHMA4 present 11 and 9 histidine stretches within their last C-terminal portion, respectively. BjHMA4 bears 14 histidines, and two contiguous Q and A residues were inserted into the middle of the histidine-rich region (Fig. 1). The highly divergent sequence of BjHMA4, especially within the C-terminal region, suggests that HMA4 was subjected to positive selection during adaptive evolution and may play a significant role in the hyperaccumulation-related characteristics of *B. juncea* [9, 14].

Previous studies have shown that the HMA4 N-terminal and C-terminal domains can function separately and can interact [12]. The N-terminal domains that are responsible primarily for transport activity require energy in the form of ATP. Evidence for this fact was provided via an altered AtHMA4D401A protein that was substituted in place of the strictly conserved ATPase sequence DKTGT, which resulted in the disruption of metal tolerance by AtHMA4 [11]. However, the position and function of the C-terminal domain remain unclear. In addition to their binding activity, C-terminal domains may participate in subcellular localization, self-chaperone activity, metal concentration perception, and regulation of transport activity. Catherine et al. demonstrated that the expression of TcHMA4-C and AtHMA4-C provided Cd tolerance but that the tolerance provided by AtHMA4-C was much lower. In contrast, overexpression of the entire *HMA4* from *Thlaspi* or *Arabidopsis* resulted in no significant difference in Cd sensitivity [12]. These findings also suggested that the N-terminal and C-terminal domains of HMA4 could function separately and that HMA4 was subjected to positive selection during adaptive evolution, especially the C-terminal region.

BjHMA4 has a relatively large number of CC dipeptides (Cys pairs) because, as occurred for BjHMA4, BjHMA4R was similarly inserted between the first two CC pairs of AtHMA4, as shown in Fig. 1. The first two CC pairs of AtHMA4 are indicated by blue numbers in Fig. 1. The first two pairs are the only conserved part of the C-terminal region of the Cd/Pb transporter AtHMA3 [18]. Catherine et al. reported that these pairs might play an important role in metal transport. However, the substitutions AtHMA4C754/5A and AtHMA4C782/3A, which affect the first two Cys pairs within the C-terminal region of the protein, had no effect on *ycf1* and *zrc1* complementation [11]. Interestingly, BjHMA4 does not present apparent transport and binding activity, but BjHMA4R exhibited strong biological activity in the present study, and we speculate that this region of HMA4 is responsible for heavy metal binding and may function as a sensor or buffer that interacts with other proteins and does not function as a self-chaperone that participates in self-transport activity [11, 19].

### The mechanism of high Cd tolerance conferred by *BjHMA4R*

The yeast strains used in the present study had distinctly different genetic backgrounds: BY4741 is the WT; *ycf1* has a mutation in an ABC transporter (YCF1 [Yeast Cadmium Factor 1], which drives Cd-diglutathione complexes to the vacuole, is disrupted); and *zrc1*, *cot1* and *zrc1&cot1* (YK44) are mutants whose CDF cation transporters ZRC1 and/or COT1 are disrupted. Thus, the Cd tolerance phenotypes could not simply be interpreted as specific functional complementation of increased ion compartmentation, as BjHMA4R also alleviates the Cd sensitivity of WT yeast under high Cd concentrations. We speculated that the common mechanism by which BjHMA4R conferred Cd tolerance to yeast cells was attributed to strong binding activity in the cytosol. The subcellular localization of BjHMA4R in yeast supported this speculation.

In the present study, we observed reduced Cd accumulations in BjHMA4R-transformed YK44 and *E. coli*; the only explanation for this phenomenon is that the expression of BjHMA4R promoted Cd efflux. This could also improve the cells’ Cd tolerance to some extent, thus prompting the growth of yeast and *E. coli*. With respect to the mechanism, we speculated that BjHMA4R is a small peptide (115 amino acids) that could interact with other metal transporters as a chaperone such as ATX1 [20, 21], resulting in increased Cd efflux. Only nonplant eukaryotes do not contain members of the P_1B_-ATPase family, so *E. coli* has HMA4 but yeast does not. We speculated that the P_1B_-ATPase family led to a greater decrease in Cd accumulation in *E. coli* than in yeast. There was no significant difference in Zn accumulation between BjHMA4R-transformed yeast cells and BL21 or control cells under 200 μM ZnCl_2_ stress, except in BjHMA4R-transformed wild-type yeast BY4741 cells, which showed slightly decreased Zn accumulation compared with control cells. These findings suggested that BjHMA4R might not bind Zn strongly under 200 μM ZnCl_2_ stress and thus would not affect Zn homeostasis in cells.

### Substrate specificity, form and binding kinetics of BjHMA4R

The expression profiles of *BjHMA4* suggested that the most suitable substrates of BjHMA4 were Zn and Cd. The yeast drop-tests indicated that the substrates of BjHMA4R were Zn and Cd, and *E. coli* drop-tests verified this. None of the *BjHMA4R*-transformed yeast strains exhibited metal tolerance under high Ni, Co or Pb concentrations. However, all of the BjHMA4R-transformed yeast strains exhibited significant and similar levels of Cd tolerance, and the *BjHMA4R*-transformed YK44 cells exhibited Zn tolerance under 1.5 mM Zn stress.

Verret et al. showed that, in contrast to YCF1, AtHMA4 does not need a glutathione S-conjugated form of Cd for transportation [11]. This finding tentatively confirmed that the entire suite of AtHMA4 transport activity did not require the glutathione S-conjugated form in the in vivo experiment but could not entirely exclude the possibility that some other small molecules participate in the transportation. Additional experiments are needed to determine the substrate form of HMA4, and we will determine the substrate form of BjHMA4R via biochemical assays in vitro.

We believe that only *BjHMA4R*-transformed YK44 exhibited Zn tolerance under low Zn concentrations because of the relatively high Zn accumulation in the cytosol of YK44 cells. ZRC1 and COT1 are both localized in the vacuolar membranes of yeast, and the disruption of both transporters weakened the Zn compartmentation into the vacuoles. Under relatively high Zn concentrations (such as 4 mM), the growth of *BjHMA4R*-transformed YK44 and control cells on solid media was completely inhibited; all of the other yeast strains (BY4741, *ycf1*, *zrc1*, *cot1*), regardless of whether expressed *BjHMA4R* or not, grew well on solid media that contained with 4 mM Zn. This case was quite different from that of Cd stress, and we proposed that the binding kinetics of Zn and Cd varied greatly and that the affinity of BjHMA4R for Cd was much higher than that for Zn. Thus, the *K*_m_ of BjHMA4R for Cd may be much lower than that for Zn, and the *V*_max_ of BjHMA4R for Cd may be much higher than that for Zn. This speculation requires the biochemical characterization of BjHMA4R in vitro, and the results of our *E. coli* experiments supported the above speculation.

On the basis of our findings, we hypothesize that BjHMA4R functions as a sensor or buffer region in BjHMA4 and that its activity is regulated by metal concentration. Functional analysis of AtHMA4 and TcHMA4 by heterologous expression in yeast yielded controversial results. We proposed that the Cd concentration used in the yeast stress experiments was the key factor that caused the opposite results. With respect to AtHMA4, the highest Cd concentration used in the yeast stress experiments was 0.12 μM [7]; the lowest, 10 μM [12]. With respect to TcHMA4, the lowest Cd concentration used in the yeast stress experiments was 75 μM [14]; the highest, 50 μM [12]. From this perspective, we can explain the seemingly paradoxical result that Zn tolerance and Zn accumulation phenotypes occurred simultaneously in the yeast strain expressing AtHMA4 under very high Zn (20 mM) [11].

The AhHMA4 C-terminus showed high Zn tolerance and low Cd tolerance. Partial peptides from the TcHMA4 C-terminus showed relatively high Cd tolerance (200 μM CdCl_2_, but the substrate specificity was not shown in this paper). BjHMA4R presented high Cd tolerance (500 and 600 μM CdCl_2_ in yeast and *E. coli*, respectively) and low Zn tolerance under limited conditions (such as in the impaired-vacuole-compartmentation yeast YK44 and in the no-vacuole *E. coli*), and BjHMA4R did not improve yeast or *E. coli* Ni, Co or Pb tolerance. Thus, the substrates of BjHMA4R may only be Cd and Zn, but it has high affinity with Cd. Moreover, considering that orthologs of BjHMA4 have a highly conserved N-terminus and more divergent C-terminus and homologous sequences to BjHMA4R only exist in Brassicaceae, we suggest that domains of the HMA4 C-terminus may play important roles in determining substrate specificity and binding affinity.

### Application prospects of BjHMA4R

BjHMA4R may have useful applications with respect to the treatment of wastewater that contains Cd by microbiological methods and enhancing the phytoremediation potential of plants via biotechnology. We are currently expressing *BjHMA4R* in *Arabidopsis* and rice plants to study their potential environmental applications; our efforts include the development of plants that exhibit increased heavy metal tolerance and possibly increased hyperaccumulation of metals. Furthermore, we are currently expressing BjHMA4R in human cells to determine whether BjHMA4R participates in the control of oxidative stress components such as metallothionein (MT).

## Conclusions

We cloned *BjHMA4* from *Brassica juncea*. The deduced amino acid sequence contains a four-repeat region, named BjHMA4R. BjHMA4R has 4 CC pairs that may be involved in heavy metal binding. Detailed characterization of BjHMA4R indicated that BjHMA4R localized in the yeast cytosol and substantially and specifically improved Cd tolerance and accumulation under low heavy metal concentrations. *E. coli* experiments verified this function. Thus, our study found a candidate gene that could be applied to the treatment of wastewater that contains Cd by microbiological methods and phytoremediation.

## Methods

### Experiments on *BjHMA4*

#### Plant materials

Seeds of Indian mustard (*B. juncea* L.) were kindly provided by the National Germplasm Resources Lab, USA (IP: 173874). Sterilized *B. juncea* L. seeds were germinated on Murashige and Skoog (MS) solid medium at 25 °C under a 16-h photoperiod. Seven days after germination, the seedlings were transferred to half-strength Hoagland solution, which was refreshed every 5 days.

The rice and wheat plants and culture conditons were identical to Tan et al. [22].

#### Gene cloning

The total RNA was extracted from the Indian mustard seedlings using TRIzol reagent (Invitrogen, San Diego, CA). cDNA was synthesized from the DNase-treated total RNA using a GIBCO BRL SuperScript First-Strand Synthesis System. The full-length coding sequence was isolated in three stages. An oligonucleotide primer designed from the most conserved 3` regions of *HMA2/4* cDNAs from *A. thaliana*, *T. caerulescens* and *A. halleri* (BjHMA4–3`: 5`-TGACATGAAACTGAAAGGTGGTTC-3`) was used for 3` rapid amplification of cDNA ends (3’RACE) (SMART RACE kit, Clontech); the 3` cDNA was sequenced and used to design another primer (BjHMA4-interR: 5`-ACAACCTGAACCACCTTT-3`). Similarly, an oligonucleotide primer was designed from the most conserved 5` regions of *HMA2/4* cDNAs from *A. thaliana*, *T. caerulescens* and *A. halleri* (BjHMA4-interF: 5`-AAGAGTTACTTCGATGTTTT-3`), after which a large partial cDNA fragment of BjHMA4 was cloned using the primers BjHMA4-interF and BjHMA4-interR. BjHMA4-specific primers designed from the above partial cDNA (BjHMA4–5`1: 5`-TCAGACGGACAACAGATT-3` and BjHMA4–5`2: 5`-CAACAGATTCCCAAAACA-3`) were used for 5’RACE (SMART RACE kit, Clontech). Last, full-length cDNA was cloned using BjHMA4-F: 5`-ACGACGCCGGACTCAAGGA-3` and BjHMA4-R: 5`-GCGTCTGAGAATCAAAGC-3` primers. The PCR products were then purified and cloned into a pMD18-T vector (Takara Bio), after which sequencing was performed.

#### Sequence analysis

HMA4 sequences were aligned using ClustalW [23] and DNAMAN 6.0; the alignment was then manually adjusted using PSBLAST pairwise alignments. Putative transmembrane domains were identified using TMHMM [24] and hydropathy analyses [25].

#### Stress treatments and quantitative real-time PCR analysis

Hydroponic Indian mustard seedlings were used for detecting *BjHMA4* expression in various tissues at different stages. The roots of seedlings treated with 600 μM ZnCl_2_ for different durations were used for measuring *BjHMA4* expression under Zn stress. Seedlings treated with CdCl_2_ (200 μM), ZnCl_2_ (2000 μM), or NiCl_2_ (400 μM) for 6 h and seedlings for which Zn, Mn and Cu supplies were absent for 1 week were used to measure *BjHMA4* expression levels in the roots and shoots under various abiotic stresses.

Real-time PCR was performed using SYBR Green (Toyobo, Osaka, Japan) with an MX3000P instrument (Stratagene Laboratories, La Jolla, CA). The *BjHMA4* primers used for real-time PCR were 5′-TCTGTGGCAAAGAAGTAA-3′ and 5′-ACCAAACTAGACGACCCT-3′, and the endogenous reference primers were those associated with the *B. juncea* actin gene (GenBank ID: KM881428.1): 5′-AATCGCTGACCGTATGAG-3′ and 5′-TCTGTTGGAAGGTGCTGA-3′.

#### Yeast transformation and Zn/cd tolerance analysis

The methods were identical to Tan et al. [22].

#### Plant plasmid construction

The methods were similar to Tan et al. [22]. The pUN1301-BjHMA4 and pLI-Act-MCS L42 rev-BjHMA4 expression constructs were generated by inserting BjHMA4 expression cassettes into the BamHI/KpnI and PstI/HindIII sites of pUN1301 (driven by the ubiquitin promoter) and pLI-Act-MCS L42 rev (driven by the rice Actin1 promoter), respectively.

#### Plant transformation

The methods were identical to Tan et al. [22].

#### Metal contents and translocation analysis

The methods were identical to Tan et al. [22].

### Experiments on BjHMA4R

#### Yeast and *Escherichia coli* cultures and transformation

The *Saccharomyces cerevisiae* reference strain BY4741 (*MAT*α *his3D1 leu2D0 met15D0 ura3D0*) and mutant strains *zrc1* (similar to BY4741 but containing *YMR243c::kanMX4*), *ycf1* (similar to BY4741 but containing *YDR135c::kanMX4*), *cot1* (strain BY4742: *MAT*α *his3D1 leu2D0 lys2D0 ura3D0 YOR316c::kanMX4*) and *end3* (similar to BY4741 but containing YNL084c::kanMX4) were obtained from Euroscarf (Frankfurt, Germany). Prof. Dietrich H. Nies (Martin Luther University, Germany) kindly provided the yeast mutant strain YK44 (*ura3–52 his3–200*, ∆zrc1, ∆cot1, *MAT*α). The cells were grown in YPD (1% yeast/2% peptone/2% dextrose; Difco), YPGAL (1% yeast/2% peptone/2% galactose; Difco), and SD (Difco; Synthetic medium with 2% dextrose) media. The *E. coli* strain BL21 was part of a commercial batch from Transgen Biotech, China. LB medium (1% tryptone/0.5% yeast extract/1% NaCl; protein expression was induced by 0.5 mM isopropyl β-D-1-thiogalactopyranoside [IPTG]) was used for growing *E. coli*.

The repeat region of *BjHMA4* cDNA (*BjHMA4R*) was obtained using BjHMA4R-specific primers (BjHMA4R-F-*Kpn*I: 5`-GGGGTACCATGAAGAAACCAAGTAGT-3` and BjHMA4R-R-*Xho*I: 5`-CCGCTCGAGTCAAGCAGTCCCCACATG-3`), and pMD18-BjHMA4 served as a template. The *BjHMA4R* cDNAs were cloned into a pYES2 vector (Invitrogen) and a pEASY-Blunt E1 expression vector (Transgen Biotech, China), which were subsequently transformed into yeast and *E. coli*, respectively, using chemical methods [26, 27].

### Subcellular localization

A pUG36 expression vector (provided by Prof. Filleur), which harbors the eGFP-BjHMA4R fusion protein, was used to observe the localization of BjHMA4R in yeast. BjHMA4R cDNA was amplified using BjHMA4R-specific primers (gfpBjHMA4R-F-*Xba*I: 5`-TGCTCTAGAATGAAGAAACCAAGTAGT-3`; BjHMA4R-R-*Xho*I: 5`-CCGCTCGAGTCAAGCAGTCCCCACATG-3`). The resulting PCR products were cloned into the XbaI-XhoI restriction sites of pUG36 for expression in yeast. The yeast strains transformed with pUG36/BjHMA4R or pUG36 were grown in liquid SD-URA/glucose (Glu) media (with or without methionine [Met]) for induction until the stationary phase. Yeast vacuolar membranes were selectively stained with FM4–64 (Catalog #: 70021, Biotium), a red fluorescent probe that has been demonstrated to selectively stain yeast vacuolar membranes [28]. The cells (1000 μL at an OD_600_ of 0.4–0.6) were incubated in 10 μL of FM4–64 (2 mg/mL) for 20 min at 29 °C, washed twice with YPD medium, incubated with shaking for 2 h in 3 mL YPD and then washed twice with SD-URA medium before observations via confocal microscopy (Zeiss LSM880). For visualization of eGFP fluorescence, excitation at 488 nm was performed with an argon ion laser, and emission signals between 500 and 535 nm were collected. For visualization of eGFP and FM4–64 colocalization, a second detector was used to collect emission signals from 650 to 800 nm [29].

### Metal tolerance tests

Yeast cells were cultured in YPD at 30 °C overnight with shaking. We used 5-μL aliquots of yeast cultures that had reached an OD_600_ of 1 to produce three tenfold serial dilutions. We used the same sterilized media for drop-test experiments on solid media. Metal solutions (ZnCl_2_, CdCl_2_, Pb(NO_3_)_2_, Ni(NO_3_)_2_ and CoCl_2_) were added to the liquid or solid media to produce different concentrations, as indicated in the figure legends. All drop-test experiments were performed in duplicate and were independently repeated at least three times. *E. coli* cells were assayed for heavy metal tolerance via LB media supplemented with ZnCl_2_ and CdCl_2_.

Yeast and *E. coli* cells were cultured in liquid YPD and LB media that contained CdCl_2_. The cells within 15 mL of a bacteria and yeast fluid were collected by centrifugation at two different time points after treatment, after which the cells were dried at 95 °C for 48 h to determine their dry weight.

### Determination of intracellular metal contents

Yeast strains were grown overnight at 30 °C in liquid YPD medium. The cells were incubated at a density adjusted to OD_600_ = 0.2 in the presence of 30 μM CdCl_2_ and 200 μM ZnCl_2_ for different durations. After incubation, the cells collected by centrifugation were washed twice with 50 mM EDTA followed by once with distilled water, after which they were dried at 95 °C for 48 h to determine their metal contents.

Cells resuspended in distilled water were mixed with an equal volume of concentrated HNO_3_, incubated at 95 °C for 2 h and then diluted with 2 volumes of distilled water. The Cd content in the cells was determined with an inductively coupled plasma trace analyzer emission spectrometer (Thermo ICAP-Q).

A similar procedure was carried out to determine the Cd and Zn accumulation in *E. coli* cells transformed with BjHMA4R clones. All the reported metal accumulation values are the means ± SEs, as determined via four replicate experiments.

### Measurement of intracellular Cd^2+^ flux in response to 30 μM CdCl_2_ stress

Net Cd^2+^ flux was measured using the non-invasive Micro-test Technology (NMT). The samples were measured in the testing solution with 30 μM Cd^2+^ after the electrodes were calibrated in two Cd^2+^ concentrations (solution I, 10 μM; solution II, 100 μM). The detailed methods were described previously [30, 31].

### Statistical analysis

Statistical analysis was performed using the statistical software package SPSS 19.0 (SPSS Science, Chicago, IL). A two-tailed *t*-test was conducted to analyze various indexes of the transgenic plants compared with the WT plants at 5 and 1% levels of probability. The experiments were repeated three times. The data are expressed as the means ± SD.

Statistical analysis was performed by one-way ANOVA using the statistical software package SPSS 19.0. Following ANOVA, Duncan’s multiple-range testwas performed to make comparisons between treatments [32].

## Additional files

Additional file 1: **Data S1.** Semiquantitative RT-PCR analysis of transgenic rice and wheat plants. **a**, **c**: Lane WT = an untransformed plant; lane number shows semiquantitative RT-PCR analysis of different transgenic plants; P, positive control. **b**, **d**: Real-time PCR analysis of *BjHMA4* in the transgenic rice and wheat. WT = an untransformed plant. Os1, Os2, and Os3 and Ta1, Ta2, and Ta3 show 3 independent transgenic rice and wheat lines, respectively. The data are expressed as the mean ± SE of three replicates; * and ** indicate significant levels at 5 and 1% (evaluated by Student’s *t* test), respectively. (JPG 216 kb)

Additional file 2: **Data S2.** Sequence alignment of AhHMA4, AtHMA4 and TcHMA4 (cited from Courbot et al. and modified). Asterisks (*) indicate the highly conserved amino acid residues in Brassicaceae. The start of the C-terminal fragment from AhHMA4 used in the yeast tolerance assay is indicated with a bracket [29]. The 384- (underlined) and 141-amino acid (shaded in blue) partial peptides from the C-terminus of TcHMA4 used in the yeast tolerance assay. (JPG 432 kb)

Additional file 3: **Data S3.** Growth of yeast cells expressing *BjHMA4R* under Ni, Co, Pb stress. BY4741, YK44, *ycf1*, *cot1* and *zrc1* transformants expressed pYES2 (negative control) and *BjHMA4R*, respectively. The cultures were adjusted to an OD_600_ of 1 and were serially diluted 10-fold in water. Five-microliter aliquots of each dilution were spotted either on nonselective YPD plates or on YPD plates supplemented with 5 mM Ni(NO3)_2_, 5 mM Co(NO3)_2_ and 10 mM Pb(NO3)_2_. After 3 days of incubation at 30 °C, the plates were imaged. The dilutions are indicated in the above figure, and three individual clones of each yeast transformant were analyzed. (JPG 10974 kb)

Additional file 4: **Data S4.** Ni, Co and Pb contents of yeast and *E. coli* expressing *BjHMA4R*. **a, b, d, e, g, h:** Yeast BY4741 and YK44 cells transformed with a pYES2 plasmid or a pYES2 plasmid that harbored *BjHMA4R* were grown in normal liquid YPD medium overnight. Then, they were supplemented with 200 μM Ni(NO3)_2_, Co(NO3)_2_ and Pb(NO3)_2_. The cells were incubated at 30 °C for 30 min, 70 min, 5 h, 10 h or 21 h. **c, f, i:** *E. coli* BL21 cells transformed with a pEASY-Blunt E1 expression plasmid or a pEASY-Blunt E1 expression plasmid that harbored *BjHMA4R* were grown in normal liquid LB medium overnight, then supplemented with 200 μM Ni(NO3)_2_, Co(NO3)_2_ and Pb(NO3)_2_. The cells were incubated at 37 °C for 10 h and 21 h. The metal contents of the samples were analyzed with inductively coupled plasma-mass spectrometry (ICP-MS). The results are the means ± SEs of four independent experiments performed with four different colonies. Different letters above the columns indicate significant differences (*P* < 0.05) between cell groups under the same stress treatment. (JPG 637 kb)

## Abbreviations

Cd: Cadmium
Co: Cobalt
HMA: Heavy metal ATPase
NMT: Non-invasive Micro-test Technology
Pb: Lead
QTL: Quantitative trait locus
WT: Wild-type
Zn: Zinc
