# Supplementary figures and images for: A repeat region from the Brassica juncea HMA4 gene BjHMA4R is specifically involved in Cd2+ binding in the cytosol under low heavy metal concentrations
Source: BMC Plant Biol. 2019 Feb 28;19:89. doi: 10.1186/s12870-019-1674-5 (PMC6394093; doi:10.1186/s12870-019-1674-5)

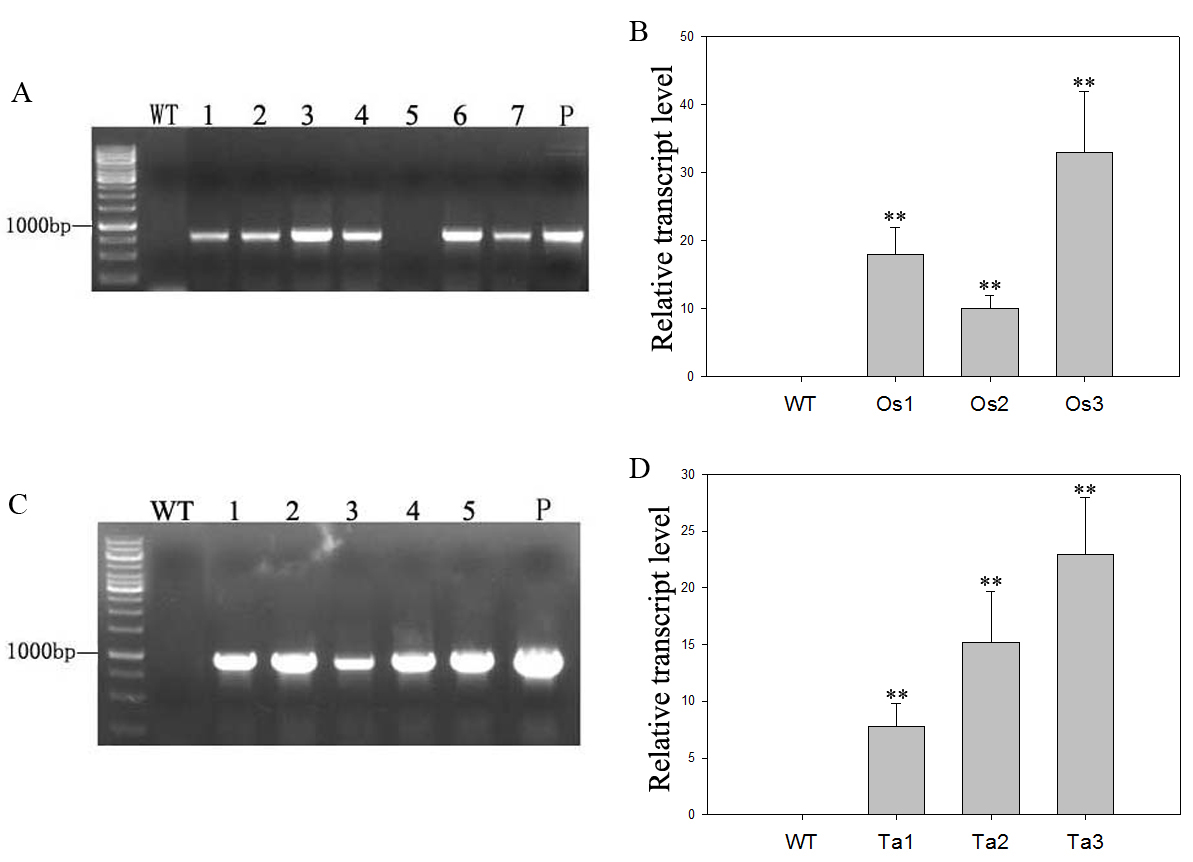

Supplement: Supplementary file 1 — Data S1. Semiquantitative RT-PCR analysis of transgenic rice and wheat plants. a, c: Lane WT = an untransformed plant; lane number shows semiquantitative RT-PCR analysis of different transgenic plants; P, positive control. b, d: Real-time PCR analysis of BjHMA4 in the transgenic rice and wheat. WT = an untransformed plant. Os1, Os2, and Os3 and Ta1, Ta2, and Ta3 show 3 independent transgenic rice and wheat lines, respectively. The data are expressed as the mean ± SE of three replicates; * and ** indicate significant levels at 5 and 1% (evaluated by Student’s t test), respectively. (JPG 216 kb) [file 12870_2019_1674_MOESM1_ESM.jpg]

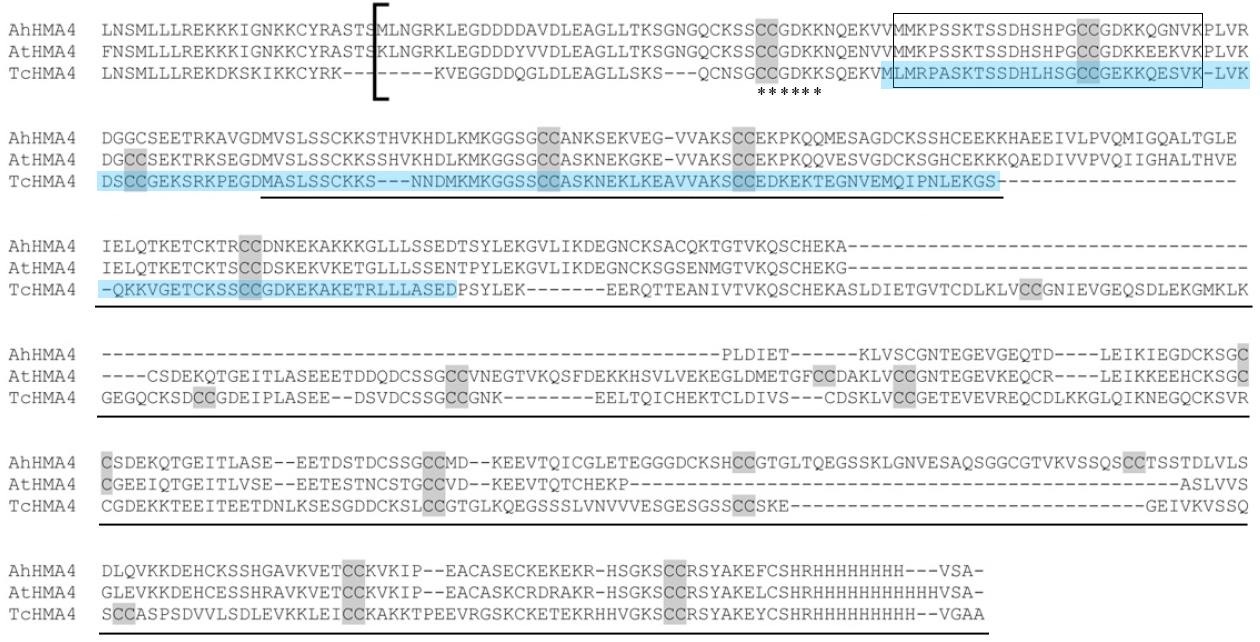

Supplement: Supplementary file 2 — Data S2. Sequence alignment of AhHMA4, AtHMA4 and TcHMA4 (cited from Courbot et al. and modified). Asterisks (*) indicate the highly conserved amino acid residues in Brassicaceae. The start of the C-terminal fragment from AhHMA4 used in the yeast tolerance assay is indicated with a bracket [29]. The 384- (underlined) and 141-amino acid (shaded in blue) partial peptides from the C-terminus of TcHMA4 used in the yeast tolerance assay. (JPG 432 kb) [file 12870_2019_1674_MOESM2_ESM.jpg]

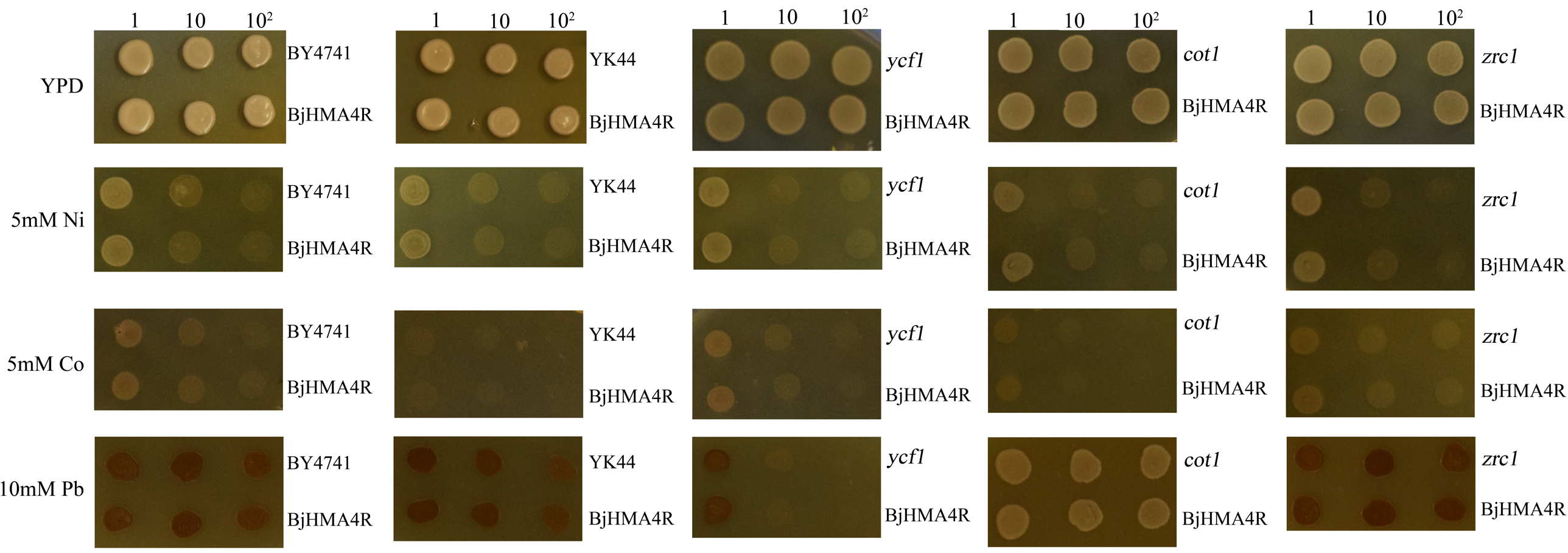

Supplement: Supplementary file 3 — Data S3. Growth of yeast cells expressing BjHMA4R under Ni, Co, Pb stress. BY4741, YK44, ycf1, cot1 and zrc1 transformants expressed pYES2 (negative control) and BjHMA4R, respectively. The cultures were adjusted to an OD600 of 1 and were serially diluted 10-fold in water. Five-microliter aliquots of each dilution were spotted either on nonselective YPD plates or on YPD plates supplemented with 5 mM Ni(NO3)2, 5 mM Co(NO3)2 and 10 mM Pb(NO3)2. After 3 days of incubation at 30 °C, the plates were imaged. The dilutions are indicated in the above figure, and three individual clones of each yeast transformant were analyzed. (JPG 10974 kb) [file 12870_2019_1674_MOESM3_ESM.jpg]

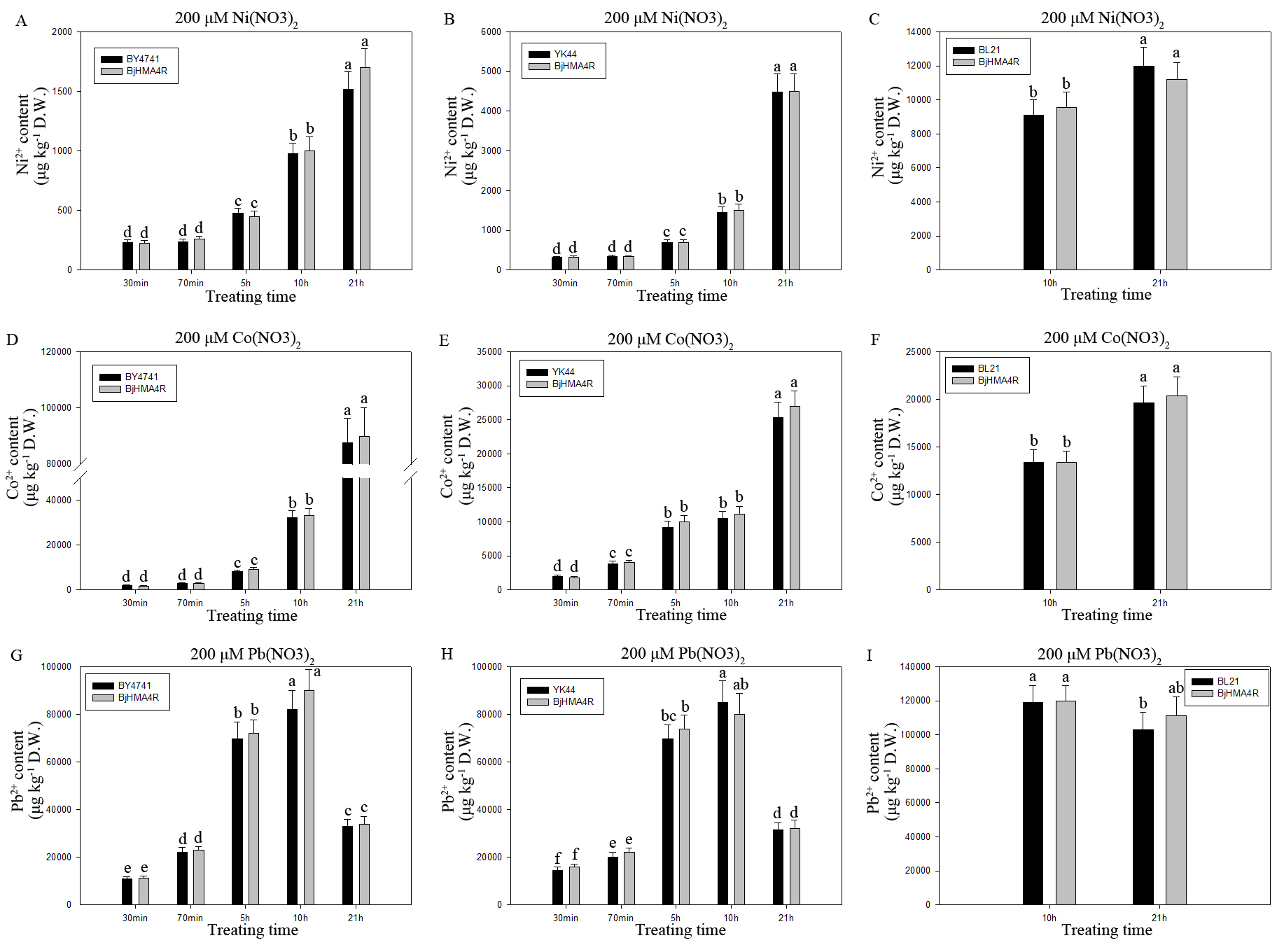

Supplement: Supplementary file 4 — Data S4. Ni, Co and Pb contents of yeast and E. coli expressing BjHMA4R. a, b, d, e, g, h: Yeast BY4741 and YK44 cells transformed with a pYES2 plasmid or a pYES2 plasmid that harbored BjHMA4R were grown in normal liquid YPD medium overnight. Then, they were supplemented with 200 μM Ni(NO3)2, Co(NO3)2 and Pb(NO3)2. The cells were incubated at 30 °C for 30 min, 70 min, 5 h, 10 h or 21 h. c, f, i: E. coli BL21 cells transformed with a pEASY-Blunt E1 expression plasmid or a pEASY-Blunt E1 expression plasmid that harbored BjHMA4R were grown in normal liquid LB medium overnight, then supplemented with 200 μM Ni(NO3)2, Co(NO3)2 and Pb(NO3)2. The cells were incubated at 37 °C for 10 h and 21 h. The metal contents of the samples were analyzed with inductively coupled plasma-mass spectrometry (ICP-MS). The results are the means ± SEs of four independent experiments performed with four different colonies. Different letters above the columns indicate significant differences (P < 0.05) between cell groups under the same stress treatment. (JPG 637 kb) [file 12870_2019_1674_MOESM4_ESM.jpg]
